# Reversing Macrophage Immunometabolic Dysfunction in Prolonged Infections Via a Glutaminolysis‐Fueled Restoration‐Based Hierarchically Selective Therapeutic System

**DOI:** 10.1002/advs.77500

**Published:** 2026-09-10

**Authors:** Weinan Yang, Zihao An, Honglu Cai, Congsun Li, Jiaxuan Zou, Wushi Cui, Wentao Chen, Tao Zhang, Shicheng Wang, Zizhan Su, Jian Xiao, Tao Sun, Bin Hu, Min Cheng, Zhongru Gou, Jianbin Xu, An Liu, Haobo Wu

**Affiliations:** ^1^ Department of Orthopedics The Second Affiliated Hospital Zhejiang University School of Medicine Hangzhou City Zhejiang Province People's Republic of China; ^2^ Orthopedics Research Institute of Zhejiang University Hangzhou Zhejiang Province People's Republic of China; ^3^ Key Laboratory of Motor System Disease Research and Precision Therapy of Zhejiang Province The Second Affiliated Hospital Zhejiang University School of Medicine Hangzhou City Zhejiang Province People's Republic of China; ^4^ Clinical Research Center of Motor System Disease of Zhejiang Province Hangzhou City Zhejiang Province People's Republic of China; ^5^ State Key Laboratory of Transvascular Implantation Devices Hangzhou People's Republic of China; ^6^ Lishui Hospital of Zhejiang University The Fifth Affiliated Hospital of Wenzhou Medical University Lishui People's Republic of China; ^7^ Bio‐nanomaterials and Regenerative Medicine Research Division Zhejiang‐California International Nanosystem Institute Zhejiang University Hangzhou People's Republic of China

**Keywords:** antibacterial immunity, drug delivery, glutaminolysis‐fueled dysfunction, infection therapy

## Abstract

Prolonged implant‐associated bone infection (IBI) fosters an immunosuppressive niche by disrupting the essential immunometabolic reprogramming required for macrophages to support antibacterial immune responses. Mechanistically, our work demonstrates that prolonged infection substantially impairs macrophage immune function by triggering a profound downregulation of glutaminase 1 (GLS1) to stifle glutaminolysis‐fueled anaplerosis. Glutaminolysis‐fueled dysfunction weakens glutamine‐derived mitochondrial reactive oxygen species (mtROS) bursts in macrophages, leading to severe deterioration of IBIs. Herein, we present a hierarchically selective therapeutic system (mGls@HEV‐MTP) for glutaminolysis‐fueled restoration in macrophages via hierarchical infection‐to‐macrophage selective GLS1 mRNA delivery. The mGls@HEV‐MTP, surface‐integrated with mannose‐functionalized antibacterial peptide (MTP) via an acid‐cleavable hydrazone bond, exploits its acid‐responsive property to decorate bacterial surfaces with mannose residues and ensure acid‐triggered prerelease of mGls@HEV for selective macrophage uptake in the acidic infection niche. This nano‐immunotherapeutic platform orchestrates bactericidal macrophage repolarization by restoring GLS1‐driven glutaminolysis‐fueled anaplerosis and simultaneously marking invading pathogens with an immune‐recognition signal, addressing both immunosuppression and poor immunogenicity in the infection niche. Notably, this work reveals the importance of glutaminolysis‐fueled anaplerosis in sustaining macrophage antibacterial immunity and provides a promising immunotherapeutic strategy for IBI eradication.

## Graphical abstract

A hierarchically selective immunotherapeutic nanoplatform, mGls@HEV‐MTP, selectively restores macrophage immunocompetence through GLS1‐mediated restoration of glutaminolysis‐fueled anaplerosis. Concurrently, it renders *Staphylococcus aureus* (*S. aureus)* more readily recognizable to the metabolically reprogrammed macrophages, thereby facilitating effective *S. aureus* clearance and remodeling the local immune microenvironment at infection sites.

## 1 Introduction

Implant‐associated bone infections (IBIs) constitute the most severe complications associated with the use of medical biomaterials in clinical settings, with *Staphylococcus aureus* (*S. aureus*) accounting for up to 50% of cases [1]. Notably, host immune dysfunction has emerged as the fundamental determinant of therapeutic failure and infection relapse, extending beyond bacterial virulence alone [2]. Even worse, prolonged IBIs pose a serious threat to individual health by exacerbating immune exhaustion [2, 3]. Macrophages, essential components of the innate immune system, recognize and engulf pathogens, followed by antigen presentation to initiate primary immune responses and secrete inflammatory cytokines to recruit additional immune cells [4, 5]. Following pathogen elimination, macrophages switch to an anti‐inflammatory phenotype and secrete growth factors to promote tissue repair and regeneration. This dynamic functional transition highlights the temporal evolution of macrophage immunobiology. However, invasive *S. aureus* changes its growth state and reduces immunogenic antigen exposure by biofilm formation, persister cell generation, and intracellular survival during infection progression, leading to prolonged or persistent infection [5]. During persistent infections, the bactericidal capability of macrophages appears substantially compromised, characterized by the impaired capacities for pathogen clearance and antigen presentation. These immunocompromised macrophages and the immunogenic antigen‐deficient niche in prolonged infection are hypothesized to impair the priming of adaptive immunity, thereby leading to immune dysfunction and infection persistence [6]. Thus, the fundamental molecular drivers that promote the shift from immune activation to profound immunosuppression warrant further investigation.

Although macrophages are reported to undergo profound metabolic reprogramming in response to adverse stimuli, the precise mechanisms and key regulatory metabolites underlying impaired macrophage functional and metabolic competence in prolonged infection remain incompletely understood [7, 8]. The amino acid metabolic status of immune cells has been reported to affect their functions in the infection niche [9, 10]. Typically, glutamine is considered a metabolic fuel for the effector functions of activated immune cells [11]. Despite multiple mechanisms underlying pathogen‐induced immunocompromise in macrophages, existing studies predominantly focus on cross‐sectional time points, lacking longitudinal comparative analyses across different infection phases. To elucidate the pathological evolution of IBIs, we analyzed different infection phases at the human, mouse, and cell levels. Through the integrated analyses of post‐infection temporal dynamics, our work elucidates that the functional integrity of bactericidal macrophages is fundamentally sustained by glutaminolysis‐fueled anaplerosis. Moreover, this metabolic flux is essential for driving the mitochondrial reactive oxygen species (mtROS) burst required for bacterial clearance and proinflammatory signal transduction. Notably, our work reveals a previously unrecognized mechanism underlying the establishment of chronic infections, in which the collapse of glutaminolysis‐fueled anaplerosis is the primary driver of host immune dysfunction as infection persists. By inducing glutaminase (GLS1) downregulation, prolonged infection stifles the mtROS burst and skews macrophages toward an immunosuppressive phenotype, characterized by PD‐L1 upregulation and compromised antigen presentation. Subsequently, the central role of this metabolic axis in macrophage immune function and IBI pathological progression is further substantiated using macrophage‐specific *Gl*s knockout mice. The pathological hallmarks of these conditional knockout mice at the acute infection phase were highly reminiscent of those observed in wild‐type mice at the chronic infection phase, characterized by significantly increased bacterial load and immunosuppressive macrophages. Overall, by defining the previously underappreciated role of glutaminolysis‐fueled anaplerosis in the pathological progression of IBIs, our work positions GLS1 as a pivotal metabolic target for therapeutic restoration of macrophage antibacterial immunity.

In light of the high protein translation efficiency and minimal risk of insertional mutagenesis [12], messenger RNA (mRNA)‐based immunotherapy is a promising strategy for guiding immunocompromised macrophages to restore glutaminolysis‐fueled anaplerosis in prolonged IBIs. Recently, lipid nanoparticles (LNPs) have emerged as a leading nanocarrier platform for therapeutic mRNA delivery due to their ionizable lipid components that facilitate mRNA encapsulation and protect mRNA from nuclease degradation [13, 14]. However, the off‐target biodistribution of LNPs limits their selective delivery within the infection niche. Inspired by the natural immune defense mechanisms against bacterial invasion, bacterial outer membrane vesicles (OMVs) have been reported to possess a powerful ability to precisely deliver therapeutic cargoes to macrophages and regulate macrophage function [15]. Due to the presence of inherited components from donor bacteria, especially pathogen‐associated molecular patterns (PAMPs), including lipopolysaccharide (LPS), peptidoglycan, and flagellin, OMVs can be effectively recognized and internalized by macrophages [16]. Therefore, the hybrid nanoparticles formed by fusing LNPs with bacterial OMVs can not only enable mRNA‐based therapy, but also be specifically sensed and phagocytosed by macrophage populations in complex infection microenvironments, thereby enabling precise modulation of macrophage immune function.

**SCHEME 1:**
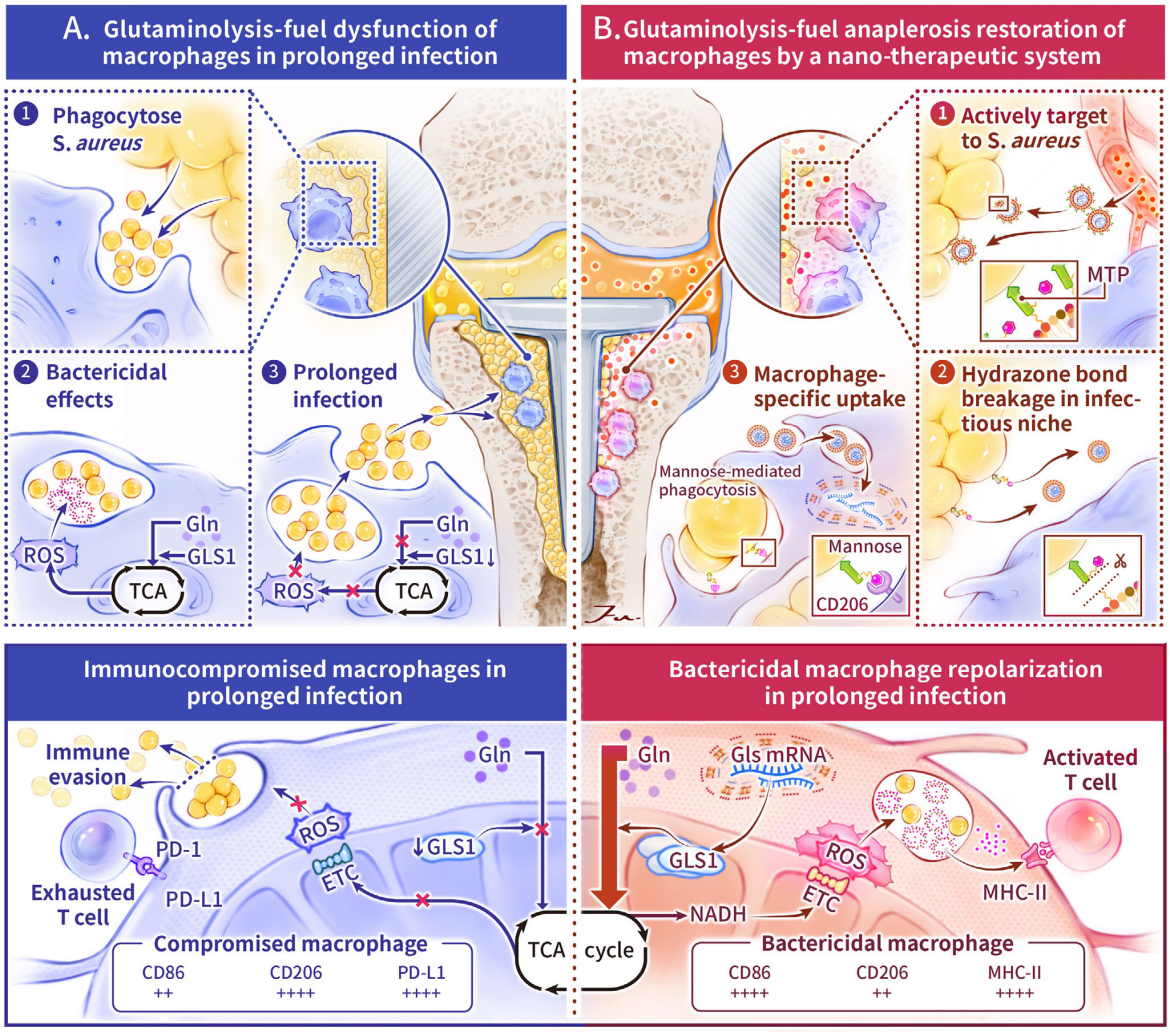
Schematic illustration of the hierarchically selective process of the therapeutic nanoplatform mGls@HEV‐MTP. The therapeutic nanoplatform is selectively internalized by macrophages at infection sites, thereby exerting dual regulatory effects within the infection niche: reprogramming macrophages into a bactericidal phenotype by GLS1 overexpression‐driven glutaminolysis‐fueled anaplerosis restoration and endowing *S. aureus* with recognizable antigens for subsequent efficient clearance by the metabolically reprogrammed macrophages, ultimately remodeling the immune microenvironment.

In this work, we uncovered the temporal dynamics of the immune microenvironment in IBIs, and revealed that GLS1 downregulation‐mediated glutaminolysis‐fueled dysfunction of bactericidal macrophages was responsible for host immunosuppression in prolonged IBIs through the integrated transcriptomic and metabolic profiles. Thus, we devised a nanotherapeutic system (mGls@HEV‐MTP) to reverse glutaminolysis‐fueled dysfunction in immunocompromised macrophages through mRNA‐mediated GLS1 overexpression. The hierarchically selective delivery system enabled impaired macrophages to be reprogrammed towards required bactericidal subsets, significantly enhancing antimicrobial efficacy and suppressing IBI progression. The dual action of mGls@HEV‐MTP, which provided the “elimination signal” (mannose residues) for bacterial clearance and restored the “metabolic engine” for antibacterial immunity, addressed both immunosuppression and poor immunogenicity in the infection niche. Overall, our work provides a scientific basis for a novel therapeutic paradigm of “metabolic reprogramming‐mediated antibacterial immunotherapy” based on GLS1‐mediated glutaminolysis‐fueled anaplerosis restoration of macrophages, and highlights its therapeutic potential for thorough eradication of *S. aureus* and prevention of infection persistence (Scheme 1).

## 2 Results

### 2.1 Single‐Cell Transcriptional and Metabolomic Profiling Identifies the Post‐Infection Temporal Dynamics of Immune Function and Metabolic Signatures in IBIs

**FIGURE 1:**
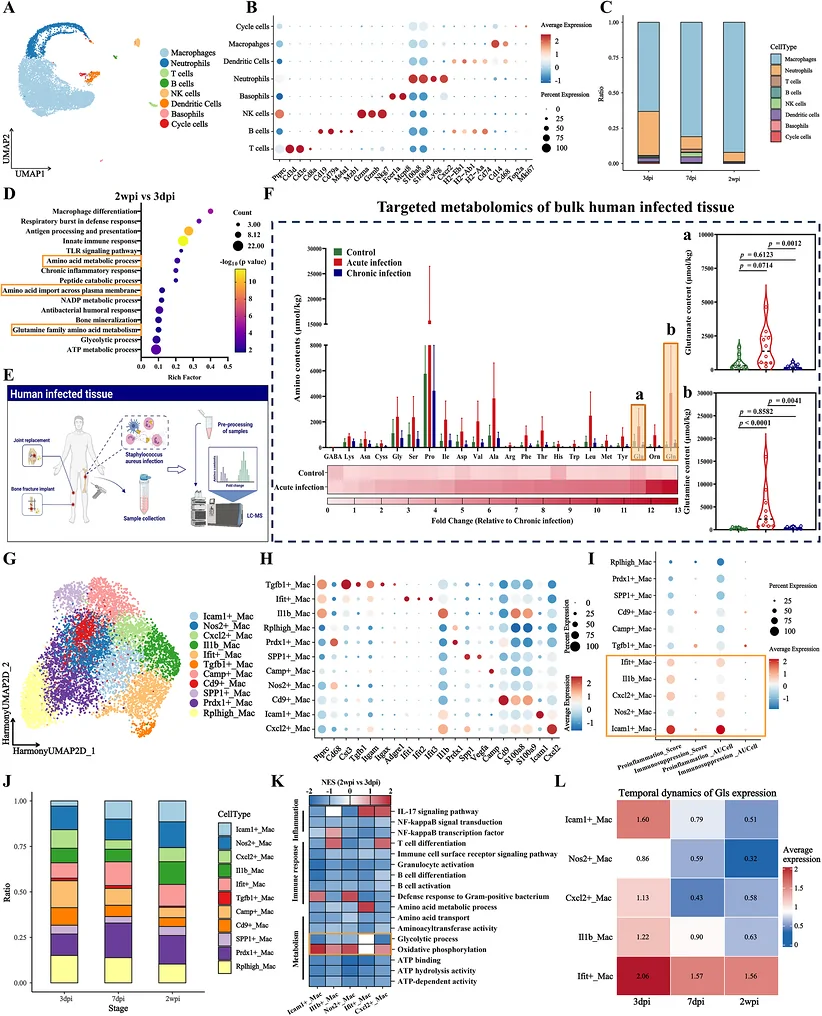
Single‐cell transcriptional and metabolomic profiling identifies the post‐infection temporal dynamics of immune function and metabolic signatures in IBIs. (A) UMAP visualization of 10 859 CD45^+^ cells from the scRNA‐seq dataset (GSE242039), stratified by infection time points (3, 7, and 14dpi). (B) Dot plot displaying canonical marker genes defining distinct cell lineages. (C) Temporal dynamics of cellular composition across cell types at 3, 7, and 14dpi. (D) GO pathway enrichment analysis of DEGs in macrophages: 2wpi versus 3dpi. (E) Schematic overview of targeted metabolomics of human infected tissue samples. Infected tissues were isolated from IBI patients (ten cases of acute infection and ten cases of chronic infection) who were diagnosed *S. aureus* monomicrobial infection via NGS or bacterial culture identification. (F) Tissue‐level contents of twenty‐two kinds of amino acids in bulk human infected tissue samples were determined by LC‐MS (*n =* 10). a and b present the relative contents of glutamate and glutamine in human IBI tissue. This figure was created by BioRender.com. (G) Subclustering analysis of macrophages revealed by UMAP projection. (H) Differential expression patterns of macrophage subcluster‐specific marker genes. (I) Proinflammatory and immunosuppressive phenotype polarization scores calculated for macrophage subclusters using established gene signatures. (J) Proportional shifts in macrophage subcluster distribution at 3, 7, and 14dpi. (K) GSEA scores of depicted pathways in proinflammatory macrophage subtypes between 3 and 14dpi. (L) Temporal expression profiles of *Gls* in proinflammatory macrophage subtypes (3, 7, 14dpi). All *n* values indicated the number of independent biological replicates. All data were presented as the mean ± standard deviation. The *p*‐values were determined by (F) the Kruskal–Wallis test with Dunn's multiple‐comparison test for comparisons among multiple groups.

To systematically characterize the landscape of IBI pathological progression, the dynamic contexture of the immune microenvironment in IBIs was investigated at the single‐cell level by analyzing publicly available datasets (GSE242039). After quality control, single‐cell RNA sequencing (scRNA‐seq) of fluorescence‐activated cell sorting (FACS)‐purified CD45^+^ cells from peri‐implant infected tissues profiled the immune cell populations and highlighted the cellular heterogeneity of the infection microenvironment in IBI mice (Figure 1A and Figure S1). Clustering analysis revealed eight cell types annotated by canonical marker genes (Figure 1B), including macrophages, neutrophils, T cells, B cells, natural killer (NK) cells, dendritic cells (DCs), basophils, and cycling cells. To identify the immune cell populations that play a dominant role in the IBI‐associated immunosuppression, the temporal dynamics of cellular composition across cell types were analyzed. Macrophages constituted the dominant immune cell populations throughout the different infection stages, and the most prominent changes in peri‐implant tissue included a significantly increased proportion of macrophages and a decreased proportion of neutrophils as infection progressed (Figure 1C and Figure S2A). These data showed that prolonged infection substantially altered the immune cell compartments in the peri‐implant tissue, where macrophages and neutrophils both showed the most significant alterations. Macrophages were capable of forming a bridge between innate and adaptive immunity against infection and regulating host immune homeostasis [5]. Thus, we sought to unveil the underlying role of macrophages in IBI pathological progression. Functional enrichment analysis of differentially expressed genes (DEGs) showed that amino acid metabolism‐related terms were enriched in macrophages (Figure 1D). In the context of metabolic reprogramming in the infection microenvironment, dysregulation of amino acid metabolism in immune cells is considered an important underlying mechanism leading to impaired antibacterial immunity [9, 17, 18]. To further characterize the alterations of amino acid metabolism during IBI pathological progression, bulk peri‐implant infected tissue samples were collected from IBI patients who were diagnosed with *S. aureus* monomicrobial infection via bacterial culture identification or Next‐Generation Sequencing (NGS) (Figure 1E). According to International Consensus Meeting on Orthopedic Infections organized by Musculoskeletal Infection Society (MSIS) criteria [19] and McPherson staging system [20, 21], 4 weeks post‐infection (4wpi) is deemed the cutoff between acute and chronic IBIs in clinical practice. Tissue‐level amino acid profiles revealed significantly increased levels of most amino acids in the infection microenvironment of acute IBIs, but amino acid levels dropped dramatically in chronic IBIs, in which glutamine (Gln) and glutamate (Glu) showed tremendous variation between acute and chronic IBIs (Figure 1F).

The chronic progression of infectious diseases is thought to be associated with macrophage reprogramming toward an immunosuppressive phenotype. PD‐L1^+^ macrophages markedly restricted T cell‐mediated adaptive immunity through inhibitory immune checkpoint interactions. Their accumulation favored the establishment of immunosuppressive microenvironments, infection persistence and relapse [22, 23, 24]. In view of the dual roles of macrophages in adaptive immune response activation and the establishment of immunosuppressive microenvironments, the heterogeneity of macrophage populations was comprehensively analyzed at single‐cell resolution. A total of 8,221 macrophages were identified across all infection stages and segregated into eleven transcriptionally distinct subsets (Figure 1G,H). Next, the transcriptional phenotypes of these macrophage subsets were examined. These macrophage subsets, consisting of Icam1^+^, Nos2^+^, Cxcl2^+^, Il1b^+^, and Ifit^+^ macrophage subsets, were characterized by immune activation and proinflammatory phenotypes (Figures S2B and S3A). Furthermore, based on defined proinflammatory or immunosuppressive gene modules and pathway activity, the aforementioned macrophage subsets (Icam1^+^, Nos2^+^, Cxcl2^+^, Il1b^+^, and Ifit^+^ macrophage subsets) more closely resembled the gene expression pattern of proinflammatory or bactericidal macrophages through dual methodologies: Seurat's AddModuleScore and AUCell algorithm (Figure 1I and Figure S3B) [25]. Next, we compared the cell composition of the macrophage subsets across different infection stages. As infection progressed, the proportion of proinflammatory macrophages notably increased from 42.5% at 3 days post‐infection (3dpi) to 60.8% at 2wpi, but of which the capabilities for stress response and immune activation were substantially limited, along with an immunosuppressive metabolic state, characterized by a metabolic bias towards decreased glycolysis and increased oxidative phosphorylation (OXPHOS) (Figure 1J,K) [26, 27]. The increased proportion of proinflammatory macrophages in prolonged infection might represent a compensatory increase in response to macrophage immune dysfunction. Accordingly, the glutamine metabolism‐associated genes of proinflammatory macrophages were significantly downregulated (Figure S4 and Figure 1L), especially with *Gls* showing the greatest fold‐change reduction. Thus, we hypothesized that glutamine metabolic dysfunction of proinflammatory macrophages might be responsible for subsequent immune dysfunction and the pathological progression of chronic IBIs.

### 2.2 Host Predisposes to Immunosuppression Associated With Glutamine Metabolic Dysfunction in Chronic IBIs

**FIGURE 2:**
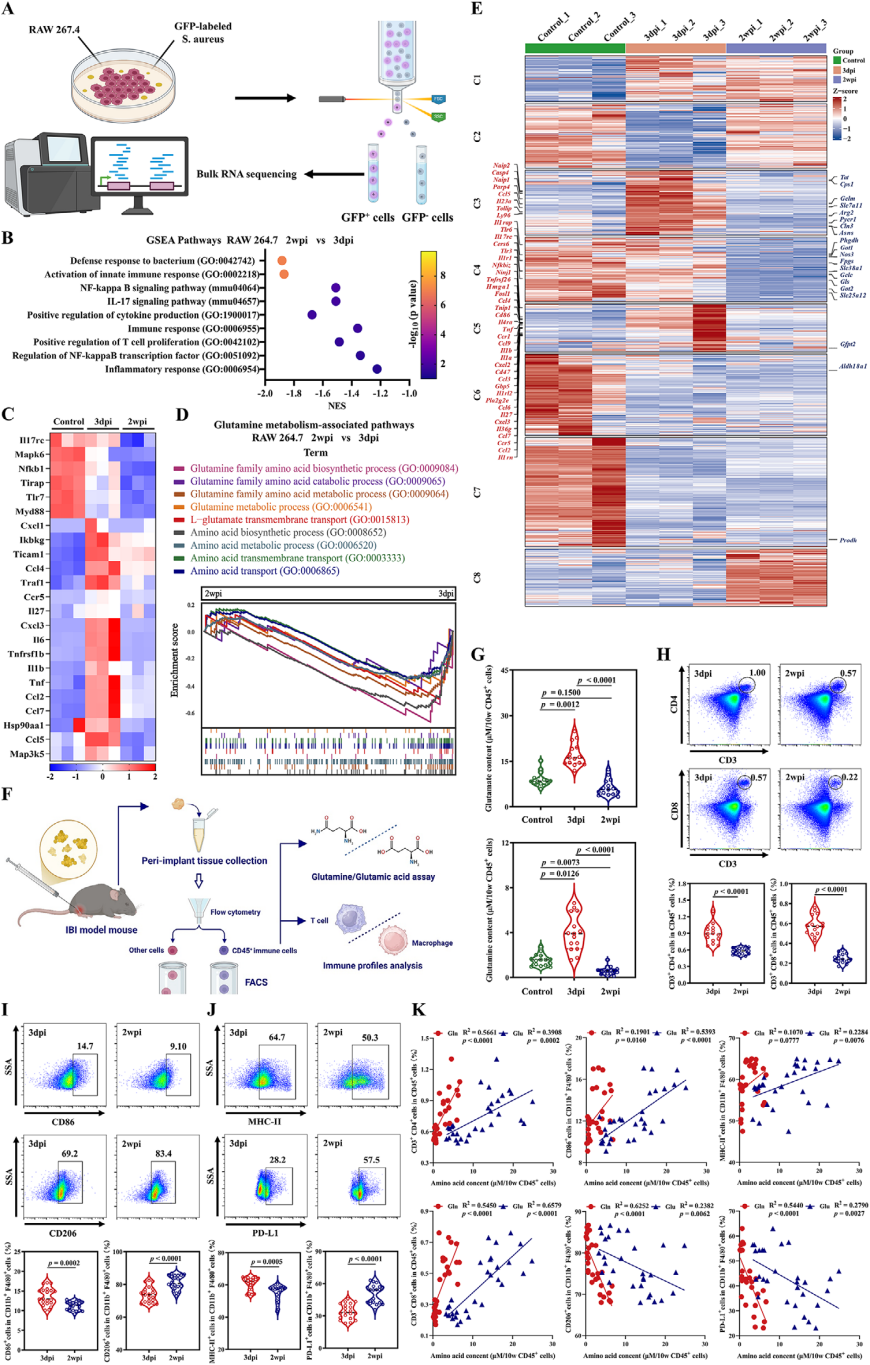
Host predisposes to immunosuppression associated with glutamine metabolic dysfunction in chronic IBIs. (A) Schematic overview of the experimental design assessing the relationship between glutamine metabolism and immunosuppression in infected macrophages. This figure was created by BioRender.com. (B) GSEA of the pathways associated with the stress response of RAW 264.7 cells to *S. aureus* infection. (C) Heatmap of transcriptional profiling depicted the DEGs involved in the stress response to *S. aureus* infection. (D) GSEA of the enriched pathways associated with glutamine metabolism in infected RAW 264.7 cells: 2wpi versus 3dpi. (E) Heatmap showing the temporal expression patterns of macrophage DEGs across different infection time points based on Mfuzz clustering analysis. DEGs were grouped into eight clusters, labeled C1–C8. Representative inflammation‐related genes are highlighted in red on the left side, whereas representative glutamine metabolism‐related genes are highlighted in blue on the right side. (F) Schematic overview of the experimental design assessing the relationship between glutamine metabolism and immunosuppression in chronic IBIsusing an IBI model mice. This figure was created by BioRender.com. (G) Quantification of intracellular glutamate and glutamine contents in CD45^+^ cells from peri‐implant bone marrow (*n =* 15). (H) Flow cytometry plots and quantification of CD4^+^/CD8^+^ T cells at infection sites (*n =* 15). (I,J) Representative flow cytometry plots and quantification of the CD86, CD206, MHC‐II, and PD‐L1 levels of macrophages at infection sites (*n =* 20). (K) The correlations between glutamate or glutamine concentrations and the ratio of CD4^+^/CD8^+^ T cells or macrophage phenotypes (*n =* 30), and *p*‐values were calculated by Pearson correlation analysis. All *n* values indicated the number of independent biological replicates. All data were presented as the mean ± standard deviation. The *p*‐values were determined by (H,I) the Student's *t* test and (J) the Mann–Whitney U test for comparisons between two groups, and (G) the Kruskal–Wallis test with Dunn's multiple‐comparison test for comparisons among multiple groups.

To corroborate the single‐cell transcriptomic and metabolomic insights and further delineate the longitudinal metabolic signatures governing macrophage dysfunction, an *S. aureus* infection model was established using RAW 264.7 macrophages for subsequent transcriptomic analysis and further experiments (Figure 2A). Initially, principal component analysis (PCA) confirmed the reliability of the sequencing results (Figure S5A). A total of more than 1,800 DEGs were identified based on the filtering criteria (absolute fold change > 2, *p*‐value < 0.05), the majority of which were functionally associated with metabolism and antibacterial immune responses (Figure S5B,C). The immune‐associated signaling pathways were inhibited as infection progressed (Figure 2B). The heatmap of DEGs related to stress response showed that *S. aureus* infection initially triggered the stress response of macrophages at 3dpi, whereas stress‐response gene expression was attenuated as infection progressed, ultimately leading to a failure of protective responses to bacterial infections (Figure 2C), which was consistent with the results of scRNA‐seq. Gene set enrichment analysis (GSEA) further revealed that the downregulated pathways associated with glutamine metabolism were more generalized at 2wpi for prolonged infection compared to 3dpi for acute infection, representing immunosuppression and persistence of *S. aureus* infection (Figure 2D and Table S1). Undeniably, the other amino acids, including valine, leucine, isoleucine, and tryptophan, also play roles in immune function [27, 28]. We screened the metabolic pathways related to several amino acids that might potentially affect immune function for GSEA analysis, including tryptophan, valine, leucine, isoleucine, arginine, serine, and methionine‐related metabolic pathways. Notably, our analyses suggested that other amino acid‐related metabolic pathways were not significantly enriched in infected macrophages across different infection phases, indicating that other amino acids might not play as important roles as glutamine in our macrophage infection model (Table S2). To examine the gene expression patterns of infected macrophages across different infection phases, we included a total of 15,153 genes across various groups at different time points. Using the factoextra and fviz_nbclust package, we analyzed these genes and determined that the optimal number for clustering was eight (Figure S5D). Then, we applied the Mfuzz algorithm to implement fuzzy clustering, dividing all genes into eight discrete gene sets, each displaying unique expression profiles (Figure S5E). Subsequently, genes related to the glutamine metabolism pathway and inflammatory response were analyzed and characterized into eight patterns. As shown in Figure 2E, most genes involved in the inflammatory response exhibited gene expression patterns of cluster 3, 4, and 5, suggesting that the gene expression pattern of infected macrophages shifted from acute infection to chronic infection, accompanied by a decline in the antimicrobial response. Genes involved in the glutamine metabolic pathway revealed convergent temporal gene expression patterns (cluster 3 and 4). Collectively, although multiple mechanisms underlying bacteria‐mediated modulation of macrophage function have been reported, existing studies have predominantly focused on cross‐sectional time points, lacking longitudinal comparative analyses across different infection phases. Therefore, the temporal dynamics of functional and metabolic signatures in macrophages following prolonged infection and the glutamine metabolic drivers of these alterations warrant systematic investigation.

To gain more insight into the relationship between immunosuppression and glutamine metabolism in vivo, we analyzed the GSE166522 dataset, which contained transcriptomic of IBI model mice at 3dpi and 2wpi to assess alterations in locoregional immune status. More than 800 differentially expressed genes (DEGs) were identified based on the filtering criteria (absolute fold change > 2, p‐value < 0.05), the majority of which were functionally associated with antibacterial immune responses (Figure S6A–C). GSEA further revealed that DEGs between different infection stages were enriched for immune‐associated signaling pathways, and antimicrobial immunity‐associated pathways showed significant downregulation (Figure S6D). Subsequent analysis of the transcriptional results revealed major alterations in the expression profiles of multiple hallmark genes involved in host immunity, including decreased expression of antigen presentation co‐stimulatory genes (Ctse, Cdldl, Dtxl, Rab4a, Wdfy4, and Ccr3), proinflammatory genes (Rasgrp1, Pnma1, Irf8, and Ifng), and T‐cell activation genes (Il2ra, Cd4, Cd8a, Btnl10, and Ermap) at 2wpi compared to 3dpi (Figure S6E). In contrast, indicative genes of immune suppression were notably upregulated at 2wpi compared to 3dpi, including PD‐L1 on antigen‐presenting cells, immune checkpoint molecules on T cells (PD‐1 and CTLA‐4), indicative genes of myeloid‐derived suppressor cells (MDSCs) (Junb, Clec4e, Ctsd, Wfdc17, and Pla2g7), and immunosuppressive cytokines (CCR1, MRC2, ARG1, and ARG2). These transcriptional data indicated phenotypic remodeling of the infection immune microenvironment into an immunosuppressive state at 2wpi, which was highly reminiscent of immune and transcriptomic profiles of chronic IBIs [29, 30]. Importantly, GSEA for the enriched pathways associated with glutamine metabolism revealed that the pathways were upregulated in defense against *S. aureus* infection at 3dpi for acute IBIs, whereas downregulated pathways were more generalized at 2wpi for chronic IBIs as the infection persisted, representing host immunosuppression and persistence of *S. aureus* infection (Figure S6F and Table S3).

Afterward, CD45^+^ immune cells were isolated from peri‐implant bone marrow of IBI model mice and uninfected mice for further experiments (Figure 2F). Consistent with the results of amino acid contents in human infected tissue samples, intracellular glutamine and glutamate contents appeared to sharply elevate at 3dpi. However, at 2wpi as the infection persisted, the levels of glutamine and glutamate decreased dramatically, even below those of uninfected CD45^+^ cells (Figure 2G). Our results demonstrated that glutamine metabolism in infected immune cells was substantially impaired in chronic IBIs, as reflected by sharply diminished intracellular glutamate and glutamine levels in prolonged infection. Furthermore, in light of the evidence indicating that downregulated glutamine metabolism in immune cells might be involved in heightened susceptibility to immune dysfunction in chronic IBIs, a more detailed analysis of the immune cell landscape was performed in the peri‐implant bone marrow cavity (Figure S7). Our flow cytometry results revealed an increased prevalence of immunosuppressive macrophages (F4/80^+^ CD206^+^ and F4/80^+^ PD‐L1^+^) and decreased infiltration of bactericidal macrophages (F4/80^+^ CD86^+^ and F4/80^+^ MHC‐II^+^) and CD4^+^/CD8^+^ T cells in peri‐implant bone marrow cavity at 2wpi compared with 3dpi (Figure 2H–J). These immune profiles demonstrated that infection led to a shift in the infection microenvironment from immune activation to immunosuppression, thereby facilitating infection persistence and immune surveillance evasion. As expected, Pearson correlation analysis showed that glutamine and glutamate contents of the isolated CD45^+^ immune cells from peri‐implant bone marrow were positively correlated with immune activation in the infection area (Figure 2K). This robust correlation between host immunocompetence and glutamine metabolism underscores a previously underappreciated mechanistic crosstalk. Thus, we speculated that glutamine metabolic dysfunction of host immune cells might be responsible for host immunosuppression as well as bacterial persistence in chronic IBIs.

### 2.3 Prolonged Infection Drives Macrophage Immunosuppressive Phenotype Polarization by Decreasing Glutaminolysis‐Fueled Anaplerosis

Currently, whether prolonged infection impairs macrophage immune function to ensure long‐term infection persistence by disrupting glutaminolysis‐fueled anaplerosis remains elusive. Therefore, to uncover the role of macrophage glutaminolysis‐fueled dysfunction in IBI progression, we further investigated this question.

**FIGURE 3:**
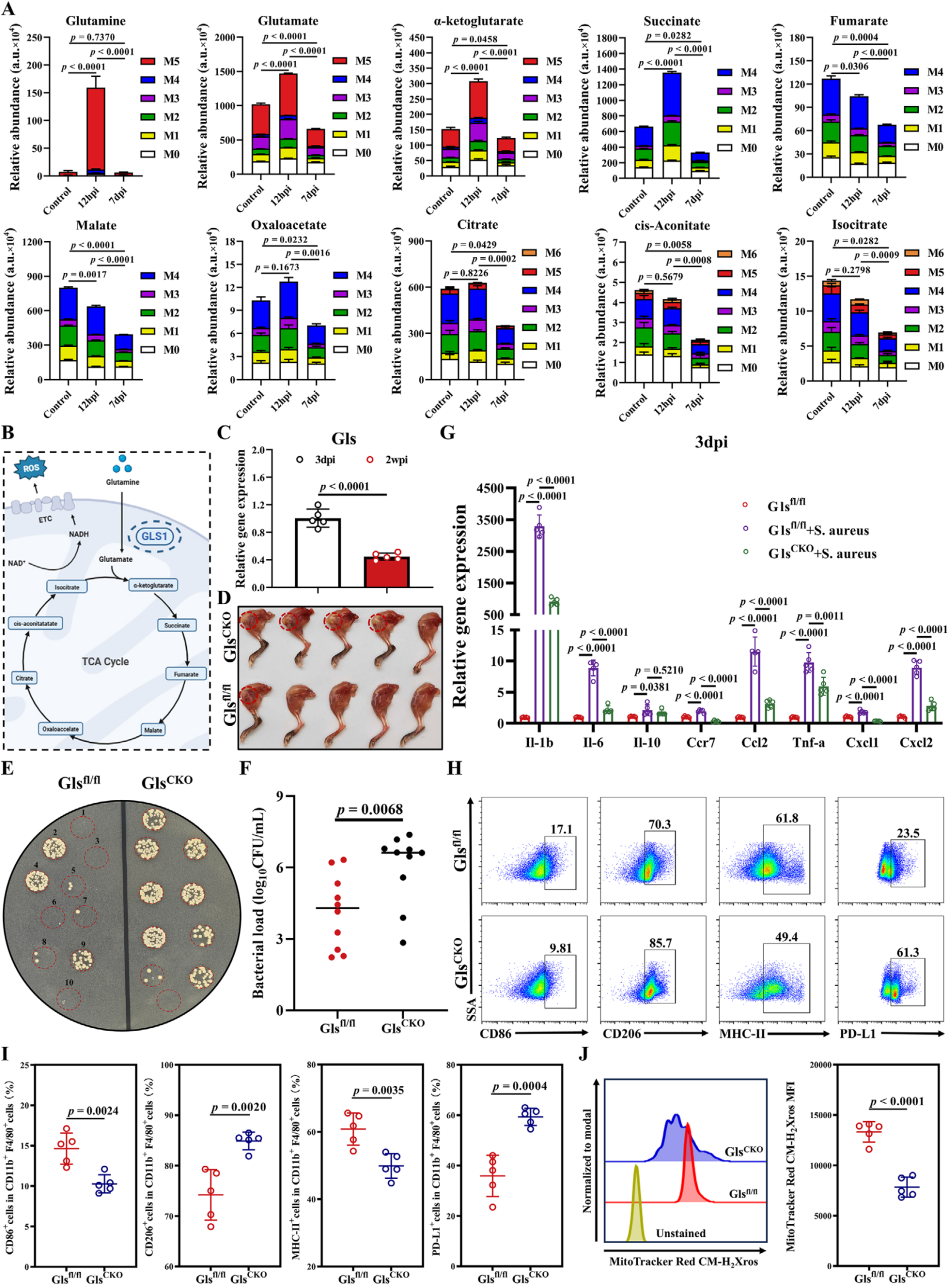
Prolonged infection drives macrophage immunosuppressive phenotype polarization by decreasing glutaminolysis‐fueled anaplerosis. (A) Mass isotopomer distribution analysis of the indicated TCA‐cycle metabolites using [U‐^13^C]‐glutamine tracing in infected macrophages treated with 8 mM [U‐^13^C]‐glutamine for 12 h (n  =  5). (B) Schematic diagram of metabolic flux regarding glutaminolysis‐fueled anaplerosis. This figure was created by BioRender.com. (C) The relative gene expression of *Gls* in peri‐implant macrophages sorted from IBI model mice at the indicated infection time points (*n =* 5). (D) Photographs of knee joints from Gls^fl/fl^ and Gls^CKO^ mice at 3dpi (*n =* 5). Red dashed boxes indicate abscess formation. (E) Culture plates and (F) corresponding bacterial load quantification of the bone marrow aspirates from Gls^fl/fl^ and Gls^CKO^ mice at 3dpi (*n =* 10). (G) qPCR analysis of proinflammatory gene expression in peri‐implant macrophages sorted from Gls^fl/fl^ and Gls^CKO^ mice at 3dpi (*n =* 5). (H) Representative flow cytometry plots and (I) quantification of the CD86, MHC‐II, CD206 and PD‐L1 levels of peri‐implant macrophages from Gls^fl/fl^ and Gls^CKO^ mice at 3dpi (*n =* 5). (J) Representative flow cytometry plots and quantification of MitoTracker Red CM‐H2Xros staining showing intracellular mtROS levels in peri‐implant macrophages sorted from Gls^fl/fl^ and Gls^CKO^ mice at 3dpi (*n =* 5). All *n* values indicated the number of independent biological replicates. All data were presented as the mean ± standard deviation. The *p*‐values were determined by (C, I, J) the Student's t test, (F) the Mann–Whitney U test for comparisons between two groups, and (A, G) one‐way ANOVA with Tukey's post hoc test for comparisons among multiple groups.

The dynamic profile of total ROS generation in infected macrophages revealed a ROS burst at 1dpi, followed by a gradual decline to a minimal ROS level at 7dpi, after which ROS generation remained at a basal level (Figure S8). Thus, 7dpi was identified as the time point at which macrophages failed to eliminate invading *S. aureus* and entered a state of persistent infection in this study. To further confirm glutaminolysis‐fueled dysfunction in infected macrophages, the [U‐^13^C]‐glutamine tracer was utilized to track glutamine‐derived metabolic flux by liquid chromatography‐mass spectrometry (LC‐MS). Glutamine flux analysis across different infection phases revealed a dynamic shift of this process in infected macrophages (Figure 3A). At 12 h post‐infection (hpi, the early proinflammatory phase), macrophages exhibited a significant increase in exogenous glutamine anaplerosis into the TCA cycle, along with increased levels of proinflammatory metabolites, such as succinate. However, at 7dpi, a phase characterized by persistent infection and dysfunctional immunocompetence, the glutamine‐derived anaplerotic flux into the TCA cycle was markedly diminished, confirming that prolonged infection led to macrophage glutaminolysis‐fueled dysfunction. To further validate the role of glutaminolysis‐fueled TCA‐cycle anaplerosis in sustaining macrophage immune function during infection, a metabolic rescue experiment was performed by supplementing BPTES‐treated macrophages with cell‐permeable dimethyl‐α‐ketoglutarate (DM‐αKG), which allowed us to bypass the GLS1‐catalyzed conversion of glutamine to glutamate and directly replenish the downstream α‐KG pool. As shown in Figure S9, the supplementation with DM‐αKG significantly enhanced intracellular bacterial clearance and mtROS burst. In parallel, DM‐αKG reversed the immunophenotypic alteration induced by BPTES and significantly increased the proportion of CD86^+^ CD206^−^ macrophages. These results confirmed that the glutamine‐derived α‐KG contributed to TCA‐cycle anaplerosis to support macrophage antibacterial function during infection.

In light of the above metabolic tracing analysis of glutamine and significantly downregulated *Gls* of proinflammatory macrophages in prolonged infections (Figure 1L), GLS1 appeared to be a pivotal regulatory node responsible for impaired macrophage immune function in prolonged infections (Figure 3B). To independently validate this transcriptomic observation, we examined *Gls* expression in peri‐implant macrophages isolated from IBI model mice at 3dpi and 2wpi. Consistent with the transcriptomic findings, *Gls* expression was markedly lower at 2wpi than at 3dpi (Figure 3C). These findings supported GLS1 as a potential metabolic regulatory node contributing to macrophage immune dysfunction during prolonged infection. Next, to investigate whether GLS1 downregulation‐mediated glutaminolysis‐fueled dysfunction promoted macrophage immunosuppression within the infection niche, we intercrossed Gls^flox/flox^ mice (Gls^fl/fl^, as control) with Lyz2‐Cre mice to successfully generate macrophage‐specific Gls‐knockout (Gls^CKO^) mice (Figure S10). We intratibially injected *S. aureus* into 8‐week‐old Gls^CKO^ and Gls^fl/fl^ mice (littermate control mice) carrying tibial titanium rod implants. At 3dpi, the mice were euthanized. We discovered that infection was markedly aggravated in Gls^CKO^ mice compared to Gls^fl/fl^ mice as indicated by photographs of knee joint abscesses (Figure 3D). The bacterial load increased by more than 100‐fold in Gls^CKO^ mice at 3dpi (Figure 3E,F). These results indicated that macrophage glutaminolysis‐fueled dysfunction played a crucial role in promoting IBI progression. Recent studies have validated that glutamine metabolism is a critical driver of a pivotal shift in mitochondrial function, which shifted from ATP synthesis to glutamine‐dependent mtROS production as an indispensable antimicrobial mechanism within macrophages [31, 32]. Adequate mtROS levels in infected macrophages, highly fueled by exogenous glutamine, were recognized as a crucial factor for bacterial clearance and proinflammatory phenotype polarization [33, 34, 35, 36]. At the acute infection phase, qPCR data revealed significantly reduced expression of proinflammatory cytokines (IL‐1β, IL‐6, IL‐10, and TNF‐α), chemokines (CCL2, CXCL1, and CXCL2), and chemokine receptors (CCR7) in GLS1‐depleted macrophages from Gls^CKO^ mice (Figure 3G). Furthermore, flow cytometry data corroborated the suppressed proinflammatory polarization and inadequate mtROS generation in GLS1‐depleted macrophages from Gls^CKO^ mice at 3dpi, suggesting the importance of GLS1‐mediated glutaminolysis‐fueled anaplerosis in macrophage immunocompetence (Figure 3H–J).

In the specific context of chronic IBIs, as revealed by our results, the scenario is further complicated by metabolic dysfunction. Prolonged infection appears to inhibit GLS1‐mediated glutaminolysis‐fueled anaplerosis, reprogramming macrophages not into a classic immunosuppressive state, but into an immunologically dysfunctional state characterized by mitochondrial dysfunction and an inability to sustain the ROS burst. Thus, reactivating glutaminolysis in the infection niche may not merely involve balancing proinflammation and immunosuppression programs, but rather involve rescuing immune cells from metabolic collapse to restore basic effector functions. Initially, we hypothesized that glutamine supplementation might help restore macrophage immune function. However, supplementation with exogenous glutamine failed to enhance total ROS and mtROS, bacterial clearance and proinflammatory phenotype polarization in infected macrophages at 4hpi and 7dpi (Figures S11A–E and S12). Furthermore, the western blot and qPCR results both suggested that GLS1 expression was not significantly upregulated in infected macrophages after treatment with exogenous glutamine (Figure S11F–G), indicating inactive glutaminolysis in macrophages even with glutamine supplementation. These data indicated that the antimicrobial immune responses of macrophages were suppressed due to impaired glutaminolysis‐fueled anaplerosis in prolonged infection.

It is acknowledged that Lyz2 is also expressed in neutrophils that play a crucial role in infection clearance. To determine whether the *Gls* deletion could impair the neutrophil antibacterial phenotype in Gls^CKO^ mice, we first examined the oxidative burst and degranulation‐associated phenotypes of peri‐implant neutrophils at 3dpi (Figure S13). Neutrophils isolated from Gls^CKO^ mice exhibited a modest but significant reduction in intracellular mtROS and total ROS compared with those from Gls^fl/fl^ mice (Figure S14A,B). However, no statistically significant difference was detected in surface CD63 levels (Figure S14C). In contrast, macrophages from Gls^CKO^ mice exhibited substantially lower intracellular mtROS, total ROS, and CD86 levels than those from Gls^fl/fl^ mice, with an approximately twofold difference (Figure S14D–F). These results indicated that the adverse effects caused by *Gls* deletion on the antibacterial phenotype of neutrophils were relatively limited compared with those in macrophages. To further exclude the contribution of neutrophils to the impaired antibacterial phenotype in Gls^CKO^ mice, we performed in vivo neutrophil depletion. The depletion efficiency of neutrophils was confirmed to exceed 96% at infection sites by flow cytometry (Figure S14G). Neutrophil depletion significantly increased the bacterial burden in Gls^CKO^ mice compared with isotype control antibody‐treated Gls^CKO^ mice, whereas it did not increase the bacterial burden in Gls^fl/fl^ mice at 3dpi, indicating that residual neutrophil bactericidal activity still contributed to bacterial clearance in the *Gls*‐deleted background (Figure S14H,I). Importantly, after neutrophil depletion, Gls^CKO^ mice continued to exhibit a significantly higher bacterial burden than neutrophil‐depleted Gls^fl/fl^ mice. These findings indicated that neutrophils contributed to antibacterial defense, but the modestly impaired neutrophil antibacterial function caused by *Gls* deletion did not fully account for the aggravated infection phenotype observed in Gls^CKO^ mice. At 3dpi, Gls deletion produced only a modest impairment in neutrophil oxidative burst, whereas peri‐implant macrophages displayed profound dysfunction in oxidative burst and bactericidal polarization. Together with the persistence of a genotype‐dependent difference in bacterial burden following neutrophil depletion, these results supported the conclusion that dysfunctional macrophages were major contributors to the overall impaired antibacterial phenotype in Gls^CKO^ mice.

Considering these findings, we speculated that guiding infected macrophages to reverse glutamine metabolic dysfunction by enhancing GLS1‐mediated glutaminolysis‐fueled anaplerosis might be critical for eradicating IBIs.

### 2.4 Establishment and Characterization of a Nanotherapeutic System for Selective Regulation of GLS1 in Macrophages

**FIGURE 4:**
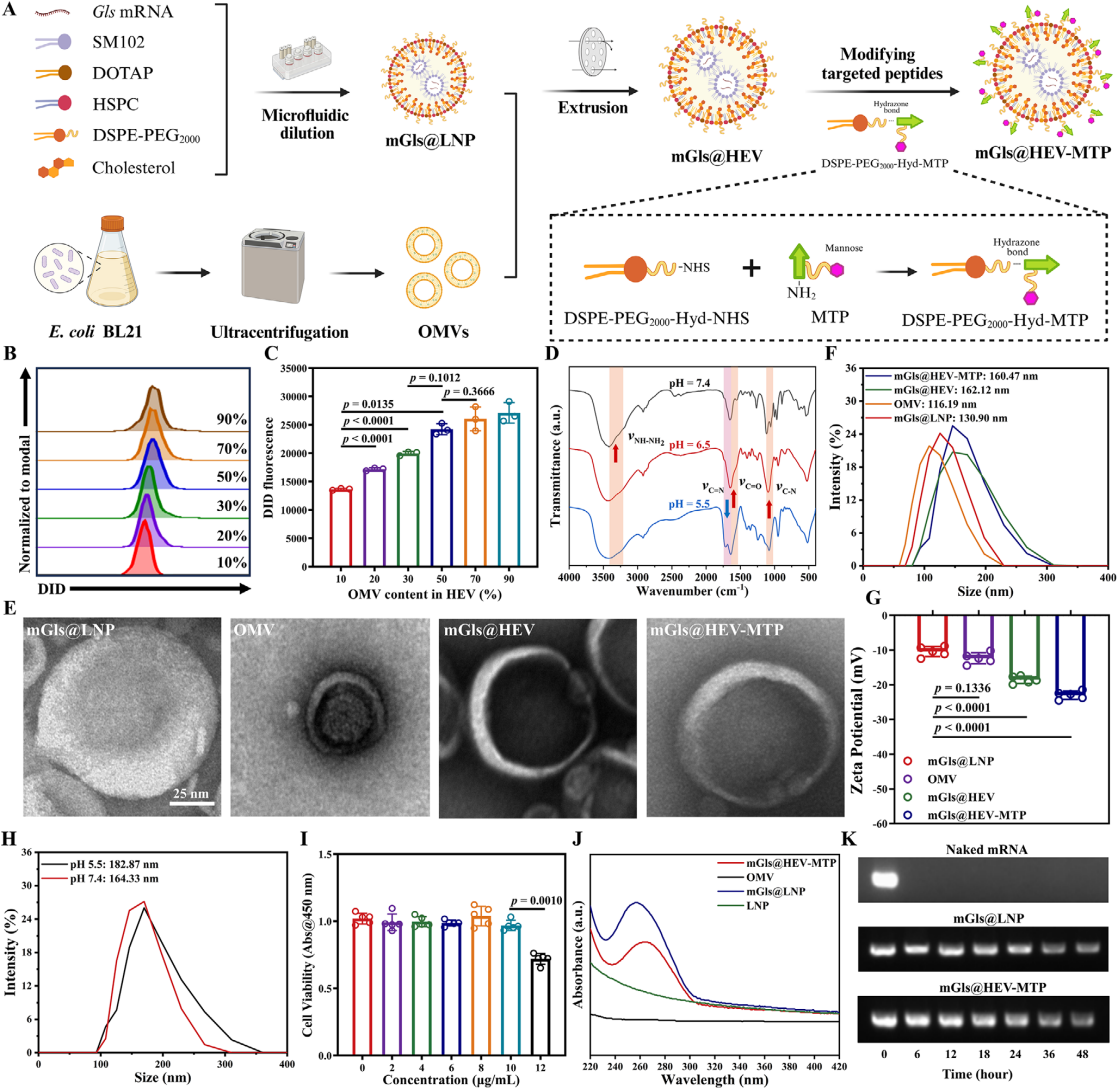
Establishment and characterization of a nanotherapeutic system for selective regulation of GLS1 in macrophages. (A) Schematic illustration of the preparation of mGls@HEV‐MTP. This figure was created by BioRender.com. (B) Representative flow cytometry plots and (C) quantification of macrophages incubated with HEV preparations of different OMV contents (*n =* 3). (D) Detection of hydrazone bond cleavage of DSPE‐PEG_2000_‐Hyd‐MTP at pH 5.5 using ATR‐FTIR. (E) Representative TEM images of mGls@LNP, OMV, mGls@HEV and mGls@HEV‐MTP nanovesicles. Scale bar, 25 nm. (F) Representative size distributions and (G) Zeta‐potential values of mGls@LNP, OMV, mGls@HEV and mGls@HEV‐MTP (*n =* 5). (H) Representative size distributions of mGls@HEV‐MTP at different pH values. (I) Cytotoxicity of mGls@HEV‐MTP in BMDMs (*n =* 5). (J) UV–vis spectra of LNP, OMV, mGls@LNP, and mGls@HEV‐MTP. (K) Electrophoretic bands of naked mRNA, mGls@HEV, and mGls@HEV‐MTP after incubation with FBS at different time points. All *n* values indicated the number of independent material sample replicates. All data were presented as the mean ± standard deviation. The *p*‐values were determined by (C, G, I) one‐way ANOVA with Tukey's post hoc test for comparisons among multiple groups.

Current clinical therapeutic strategies for IBIs typically include long‐term antibiotic administration and surgical treatments, which fail to restore immunocompetence or even exacerbate immune dysfunction, leaving patients at high risk of infection recurrence [37]. Our findings have shown that the regulation of glutaminolysis‐fueled anaplerosis is essential for the activation of antibacterial immunity, and GLS1 is a pivotal target for effective therapeutic intervention in chronic IBIs. To date, mRNA‐based therapies have emerged as promising alternative platforms for delivering therapeutic proteins [12]. Precise drug delivery and minimization of off‐target effects are essential for advancing the clinical potential of nucleic acid therapeutics. Thus, to precisely regulate GLS1 expression in macrophages in the infection niche, we developed a hierarchically selective therapeutic system that integrates infection‐specific localization with macrophage‐selective gene modulation utilizing mRNA‐based gene therapy strategy through a multi‐step process (Figure 4A).

First, *Gls* mRNA‐loaded LNPs (mGls@LNPs) were synthesized using a microfluidic device. The encapsulation efficiency of mGls@LNPs was approximately 93.1%. In view of the strong macrophage‐selective ability of bacteria‐derived outer membrane vesicles (OMVs) [15], we extracted OMVs that were derived from genetically engineered hypoendotoxic *Escherichia coli* (*E. coli*) BL21 (ΔmsbB) constructed via CRISPR‐mediated gene knockout [38, 39, 40]. Next, we fused mGls@LNPs with OMVs through co‐extrusion to obtain *Gls* mRNA‐loaded hybrid vesicles (mGls@HEVs) for macrophage‐specific delivery. To evaluate the optimal OMV hybridization ratio, OMV protein mass contents ranging from 10% to 90% were used to generate HEVs. Next, macrophages were co‐incubated with DID‐labeled HEVs with different OMV contents, followed by flow cytometry analysis. It was observed that HEVs containing more than 30% OMVs exhibited efficient delivery to macrophages (Figure 4B,C). Thus, an OMV content of 30% was selected for the synthesis of mGls@HEVs. The functionality of the immune system relies on interactions between receptors and ligands [41, 42]. In chronic IBIs, bacteria generate a niche of immune desert through poorly immunogenic antigen exposure, as a result of which macrophages fail to efficiently recognize and engulf bacteria for the activation of adaptive immunity in such an immunologically cold microenvironment [43]. Therefore, immune labeling techniques, which provide specific antigens or binding sites to enhance the selective recognition capability of immune cells, are applied in this study to achieve selective nanovesicle delivery to the infection site and to label bacteria with recognizable antigen markers. A mannose‐functionalized targeting peptide (MTP) containing the CRLYLRIGRR sequence was synthesized and was confirmed to effectively target *S. aureus* [44]. It has been reported that functionalized mannose multivalently binds to mannose receptors on macrophages and further facilitates the immune recognition and phagocytosis of bacteria [45, 46]. Next, to achieve acidic infection niche‐triggered prerelease of mGls@HEV, the triblock polymer DSPE‐PEG_2000_‐Hyd‐MTP was designed (Figure S15A). The successful synthesis of DSPE‐PEG_2000_‐Hyd‐MTP was confirmed by proton nuclear magnetic resonance (^1^H NMR) (Figure S15B). The hydrazone bond in DSPE‐PEG_2000_‐Hyd‐MTP conferred pH‐responsive properties, and the hydrazone bond cleavage in DSPE‐PEG_2000_‐Hyd‐MTP at pH 5.5 was confirmed through Attenuated Total Reflection Fourier Transform Infrared (ATR‐FTIR) spectroscopy, as a previous study reported [47]. After 24 h, the absorption peak at 1691 cm^−1^ corresponding to the *ν*_C = N_ stretching vibration decreased, accompanied by the simultaneous appearance of the *ν*_NH−NH2_ stretching vibration at 3300–3200 cm^−1^, indicating the gradual cleavage of hydrazone bond and exposure of hydrazine bond (Figure 4D). Besides, the presence of *ν*_C = O_ stretching vibration at 1650 cm^−1^ and *ν*_C−N_ stretching vibration at 1110 cm^−1^ further confirmed the release of MTP. Finally, DSPE‐PEG_2000_‐Hyd‐MTP was inserted into mGls@HEV to generate mGls@HEV‐MTP. To experimentally verify the successful membrane fusion between OMVs and LNPs, we performed a comparative protein analysis via sodium dodecyl sulfate‐polyacrylamide gel electrophoresis (SDS‐PAGE). The electrophoretic profile demonstrated that the HEVs retained protein components derived from the OMVs, which were absent in the bare LNP control (Figure S16A). These results confirmed the successful fusion of OMVs and LNPs, resulting in hybrid vesicles that incorporate both structural and proteinaceous elements from their parent components. Transmission electron microscopy (TEM) images revealed that mGls@LNP, OMV, mGls@HEV and mGls@HEV‐MTP nanovesicles all exhibited distinct lipid bilayer structures and presented saucer‐like architectures (Figure 4E). The average hydrodynamic size of LNPs was approximately 160.47 nm with a range of 92.89–267.2 nm (Figure 4F). The average zeta potential values of mGls@LNP (approximately −10.42 mV), OMV (approximately −12.38 mV), mGls@HEV (approximately −18.58 mV) and mGls@HEV‐MTP (approximately −22.97 mV) were all negative (Figure 4G). In addition, almost no alterations were observed in the particle size of mGls@HEV‐MTP after incubation in DMEM containing 10% FBS at both pH 7.4 and 5.5 for 24 h, indicating that mGls@HEV‐MTP maintained its structural integrity under acidic conditions, typical of extracellular pH in the infection niche (Figure 4H). Furthermore, the stability of mGls@HEV‐MTP in DMEM containing 10% FBS was assessed over 7 days. Daily measurements confirmed excellent particle size stability (Figure S16B). Given that the presence of nucleic acid itself can potentially enhance innate immune activation independent of the encoded protein, it is important to distinguish mRNA encoding protein‐dependent immune activation from nonspecific mRNA‐associated immune stimulation [48]. Therefore, a noncoding scrambled mRNA control was designed to distinguish nonspecific mRNA‐associated immune stimulation. To directly evaluate the intrinsic immunostimulatory activity of HEV‐MTP (loading with a noncoding scrambled mRNA control), macrophages were treated with increasing concentrations of HEV‐MTP, and the expression of the pro‐inflammatory polarization‐associated markers iNOS and CD86 was assessed by flow cytometry. Treatment with HEV‐MTP at 6 or 8 µg/mL did not significantly alter intracellular iNOS expression compared with the untreated control group (Figure S17A). In contrast, exposure to 10 or 12 µg/mL significantly increased iNOS levels, with the highest signal observed at 12 µg/mL, indicating a concentration‐dependent induction of iNOS at high doses. A similar dose‐dependent pattern was observed for surface CD86 expression (Figure S17B). These results validated that HEV‐MTP has no significant intrinsic immunostimulatory activity at concentrations below 8 µg/mL. Cytotoxicity of mGls@HEV‐MTP in vitro was evaluated using a CCK‐8 assay by incubating bone marrow‐derived macrophages (BMDMs) with mGls@HEV‐MTP at different concentrations for 24 h. At a concentration of 12 µg/mL, mGls@HEV‐MTP exhibited cytotoxicity (cell viability of approximately 76%), as a result of which 6 µg/mL was selected for further experiments (Figure 4I). Also, mGls@HEV‐MTP and mGls@LNP both exhibited the characteristic absorption peak of mRNA (approximately 260 nm) in the UV–vis absorbance spectra, indicating successful mRNA loading (Figure 4J). As illustrated in Figure 4K, mGls@LNP and mGls@HEV‐MTP both retained detectable electrophoretic bands after incubation for 48 h, in stark contrast to the nearly complete degradation of naked mRNA after 6 h of incubation, demonstrating that LNP encapsulation effectively shielded mRNA from serum nuclease‐mediated degradation and OMV hybridization did not compromise mRNA stability.

### 2.5 mGls@HEV‐MTP‐Mediated Hierarchically Selective Delivery

**FIGURE 5:**
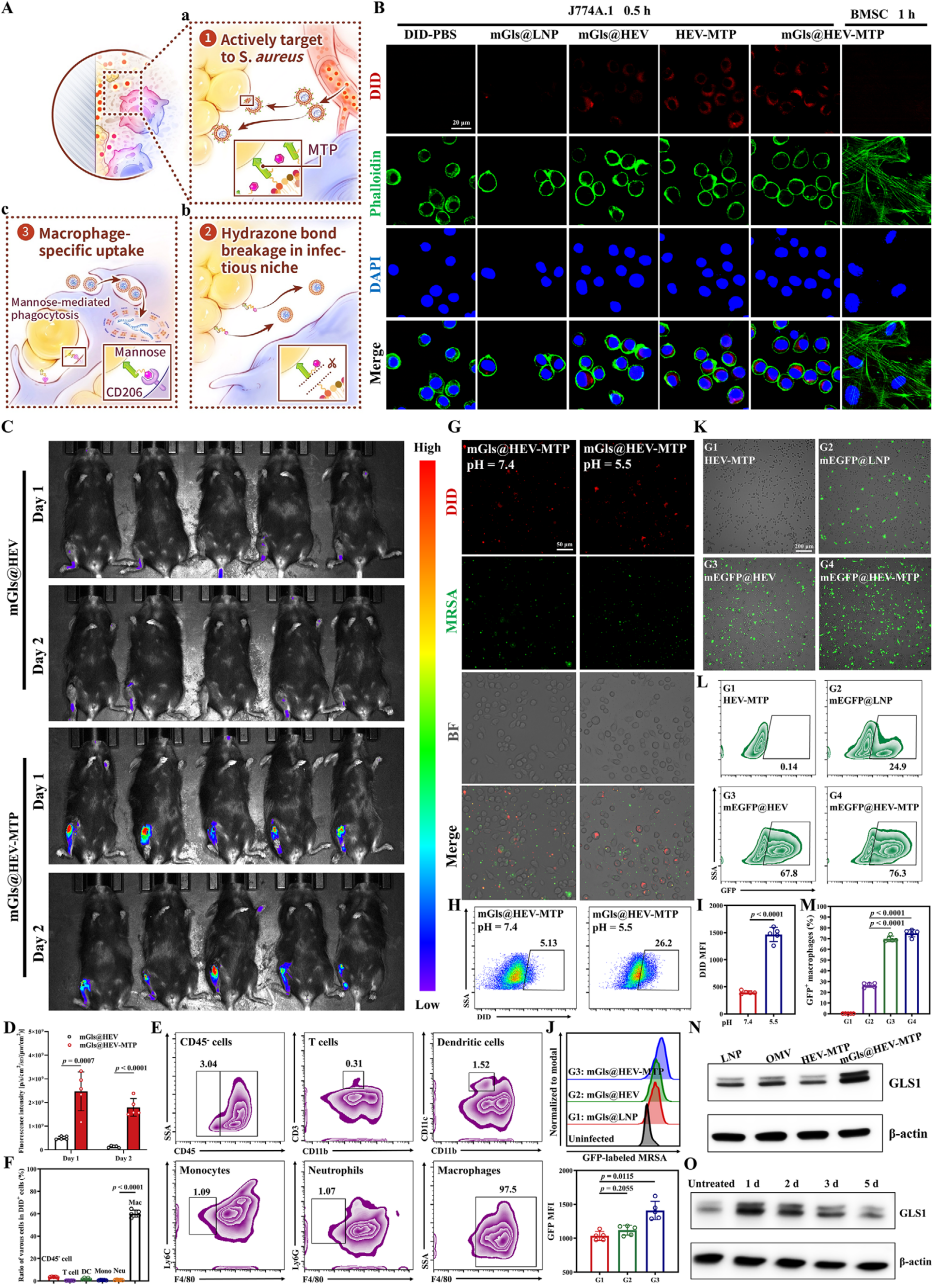
mGls@HEV‐MTP‐mediated hierarchically selective delivery. (A) Schematic illustration of the hierarchical infection‐to‐macrophage selective delivery process and mannose‐mediated bacterial phagocytosis induced by mGls@HEV‐MTP. a, the mGls@HEV‐MTP efficiently targets infection sites via intravenous injection. b, the hydrazone bond cleavage and mGls@HEV release in the acidic infection niche. c, mannose‐mediated bacterial phagocytosis via MTP left on bacterial surfaces. (B) Representative confocal images showing the targeting and internalization of the indicated nanovesicles after co‐incubation with macrophages for 0.5 h and BMSCs for 1 h. Nanovesicles were labeled with DID (red), macrophages with phalloidin (green), and nuclei with DAPI (blue). Scale bar, 20 µm. (C) In vivo fluorescence images of IBI model mice after 1 d and 2 d post‐injection of DID‐labeled mGls@HEV and mGls@HEV‐MTP. (D) Fluorescence intensity of mGls@HEV and mGls@HEV‐MTP at infection sites (*n =* 5). (E) Representative flow cytometry plots and (F) quantification showing the percentage of various cell populations (macrophages, neutrophils, monocytes, dendritic cells, T cells, and CD45^−^ cells) gated on DID^+^ cells at infection sites (*n =* 5). (G) Fluorescence images depicting the state before and after adjusting pH from 7.4 to 5.5 in the mixed solution containing DID‐labeled mGls@HEV‐MTP (red), GFP‐labeled MRSA (green) and macrophages after co‐incubation for 5 min. Scale bar, 50 µm. (H) Representative flow cytometry plots and (I) quantification of DID MFI in J774A.1 cells after 5 min of incubation with DID‐labeled mGls@HEV‐MTP and MRSA at pH 7.4 and pH 5.5 (*n =* 5). (J) Representative flow cytometry plots and quantification of the phagocytosis rate of J774A.1 cells after incubation with GFP‐labeled MRSA pretreated with mGls@HEV‐MTP at pH 5.5 (*n =* 5). (K) Representative fluorescence images showing the expression of EGFP in J774A.1 cells treated with different nanovesicles. Scale bar, 200 µm. (L) Representative flow cytometry plots and (M) quantification showing EGFP expression in macrophages after co‐incubation with different nanovesicles for 24 h (*n =* 5). (N) WB analysis of GLS1 protein expression in IL‐4‐polarized macrophages after treatment with different nanovesicles. β‐actin served as the loading control. (O) WB analysis of GLS1 protein expression in IL‐4‐polarized macrophages over time after treatment with different nanovesicles. β‐actin served as the loading control. All *n* values indicated the number of independent biological replicates. All data were presented as the mean ± standard deviation. The *p*‐values were determined by (D,I) the Student's t test for comparisons between two groups and (F, J, M) one‐way ANOVA with Tukey's post hoc test for comparisons among multiple groups.

According to our design, mGls@HEV‐MTP first accumulated at infection sites via systemic circulation and firmly bound to *S. aureus* after injection. Upon acidic infection niche‐triggered dissociation of hydrazone bond, the released mGls@HEV was selectively phagocytosed by macrophages, thereby delivering *Gls* mRNA to induce GLS1 overexpression. Simultaneously, the MTP was left on the *S. aureus* surface to endow it with recognizable antigen for subsequent efficient clearance by metabolically reprogrammed macrophages in the infection niche (Figure 5A).

To assess the uptake of DID‐labeled nanovesicles by macrophages, we co‐incubated the indicated nanovesicles with macrophages and assessed their internalization using confocal microscopy at 0.5 h. Besides, bone marrow mesenchymal stem cells (BMSCs), as a major cell population within bone, were isolated from bone marrow and co‐incubated with the indicated nanovesicles for 1 h. In this experiment, a DID‐PBS control group was included, in which PBS was treated with DID under the same conditions as the nanovesicles, to eliminate potential interference from residual DID uptake by macrophages during the nanovesicle‐labeling process. Our results demonstrated that the internalization of mGls@HEV and mGls@HEV‐MTP by macrophages was significantly higher than that of mGls@LNP (Figure 5B). Notably, compared with BMSCs, mGls@HEV‐MTP exhibited superior macrophage selectivity, which was attributed to the efficient OMV uptake capacity of macrophages. To further quantify the kinetics of nanovesicle uptake, we performed flow cytometry analyses at multiple time points, measuring the fluorescence intensity of DID‐labeled nanovesicles internalized by macrophages. Our findings revealed that mGls@HEV‐MTP uptake increased progressively over time, and mGls@HEV‐MTP uptake gradually declined after 12 h (Figure S18). Nevertheless, even at 24 h, the macrophage uptake of mGls@HEV was lower than that of mGls@HEV‐MTP. The enhanced uptake of mGls@HEV‐MTP might be attributed to mannose‐specific binding to CD206 on macrophages, facilitating more efficient internalization. To investigate the targeting capability of mGls@HEV‐MTP towards *S. aureus*, we co‐incubated GFP‐labeled MRSA with DID‐labeled mGls@HEV‐MTP for 30 min. In the mGls@HEV‐MTP group, the red fluorescence was clearly clustered and accumulated around the green fluorescence (Figure S19A). This aggregation was attributed to the targeting effect of MTP towards *S. aureus*, leading to the accumulation of mGls@HEV‐MTP around the bacterial cells. In contrast, mGls@HEV showed a diffuse and non‐specific distribution. The fluorescence colocalization analysis further demonstrated strong colocalization between mGls@HEV‐MTP (red) and *S. aureus* (green), and the Pearson coefficient reached 0.9698, indicating tight binding (Figure S19B). In the control group, no obvious binding was observed, and the Pearson coefficient was only 0.0776, confirming that mGls@HEV‐MTP possessed effective bacterial targeting capability. To further explore the infection tropism of mGls@HEV‐MTP in vivo, DID‐labeled nanovesicles were injected intravenously into the tibial IBI mouse model. As shown by in vivo live imaging, mGls@HEV‐MTP exhibited rapid accumalation at infectious sites at 1 day post‐injection, with fluorescence intensity significantly higher than that of mGls@HEV (Figure 5C,D). Notably, the fluorescence signal remained detectable at 2 d post‐injection, confirming the enhanced infection tropism of mGls@HEV‐MTP as illustrated in Figure 5A (a, Step 1). To precisely determine the biodistribution of mGls@HEV‐MTP among major cell populations in the infection niche, bone marrow was extruded to obtain single‐cell suspensions for flow cytometry analysis. This analysis quantified the proportion of CD45^−^ cells, T cells (CD45^+^ CD11b^−^ CD3^+^), dendritic cells (DCs, CD45^+^ CD11b^−^ CD11c^+^), monocytes (CD45^+^ CD11b^+^ F4/80^−^ Ly6C^+^), neutrophils (CD45^+^ CD11b^+^ F4/80^−^ Ly6G^+^) and macrophages (CD45^+^ CD11b^+^ F4/80^+^) in DID‐positive cells by the corresponding flow cytometry gating strategy (Figure S20). Within the CD45^+^ CD11b^+^ DID^+^ cells, macrophages accounted for approximately 91.6%. After normalization to the total DID‐positive population, macrophages constituted approximately 60.12% of all DID‐positive cells, while the proportions of other phagocytes in DID‐positive cells such as DCs, monocytes, and neutrophils, accounted for approximately 1.93%, 0.66%, and 0.82%, respectively (Figure 5E,F). Given that the cellular composition of the DID‐positive cell population was also influenced by the baseline abundance of cell populations in the infection sites, we analyzed the DID abundance within each cell population. The DID abundance in macrophages was significantly higher than that in each of the other analyzed cell populations (Figure S21). The results revealed that macrophages were the predominant immune cell population internalizing mGls@HEV‐MTP. To evaluate the systemic biodistribution and potential off‐target accumulation of the nanoplatform, major organs and the infection sites were collected for ex vivo fluorescence imaging after intravenous administration of the DID‐labeled mGls@HEV‐MTP for 72 h. Strong and consistently detectable fluorescence signals were observed at the infection sites in all mice (Figure S22A). Notably, the infection‐specific fluorescence intensity of mGls@HEV‐MTP was approximately 6.6‐fold higher than the fluorescence intensity of major organs at 72 h (Figure S22B), confirming the infection tropism and retention of mGls@HEV‐MTP.

To further validate the capacity of mGls@HEV‐MTP for hierarchically selective mRNA delivery to macrophages, we established a co‐culture system of macrophages and *S. aureus* to simulate the infection microenvironment and incubated the co‐culture system with mGls@HEV‐MTP. Representative fluorescence images visualized the intact hierarchically selective delivery process (Figure 5G). At pH 7.4, mGls@HEV‐MTP first bound to the surface of *S. aureus* 5 min after addition to the co‐culture system due to the affinity of MTP for *S. aureus*. As illustrated in Figure 5A (b, Step 2), when the pH dropped to 5.5, the hydrazone bond was cleaved in response, and the released mGls@HEV nanovesicles were gradually phagocytosed by macrophages. Flow cytometry analysis showed significantly enhanced uptake of nanovesicles under the acidic microenvironment, with phagocytosis rates increasing from 5.13% to 26.2% (Figure 5H,I). To validate that MTP left on the bacterial surface endowed *S. aureus* with a recognizable signal for macrophage phagocytosis after hydrazone bond cleavage, as illustrated in Figure 5A (c, Step 3), we pretreated *S. aureus* with the indicated nanovesicles under acidic conditions and then incubated *S. aureus* with macrophages for 1 h. Flow cytometry data showed significantly increased phagocytosis of bacteria in the mGls@HEV‐MTP group (Figure 5J). These results demonstrated that the design of DSPE‐PEG_2000_‐Hyd‐MTP facilitated bacterial recognition, macrophage‐mediated bacterial phagocytosis, and macrophage uptake of nanovesicles within the acidic infection niche.

As a proof of concept, we engineered an EGFP mRNA reporter system (mEGFP@HEV‐MTP) and demonstrated substantial EGFP expression in treated macrophages (Figure 5K), confirming successful mRNA delivery by our nanovesicles and subsequent intracellular translation. In addition, flow cytometry further revealed higher EGFP expression (an average level of 75.02%) in macrophages after treatment with mEGFP@HEV‐MTP compared with treatment with mEGFP@LNP (an average level of 26.62%), which might be attributed to the strong macrophage‐specific targeting ability of HEV to promote macrophage uptake (Figure 5L,M). Given that IL‐4‐polarized macrophages exhibit an immunosuppressive phenotype with CD206 expression, we utilized IL‐4‐polarized macrophages as target cells to assess the ability of mGls@HEV‐MTP to restore GLS1 expression in immunocompromised macrophages. Furthermore, we prepared three types of nanovesicles as negative controls to exclude potential interference from nano‐delivery systems with macrophage metabolism. After treating immunosuppressive macrophages with these nanovesicles, GLS1 expression levels were measured in cell lysates using Western blot (WB) analysis. Through quantitative analysis, the GLS1 expression levels in macrophages treated with mGls@HEV‐MTP increased by approximately tenfold (Figure 5N). Encouragingly, mGls@HEV‐MTP induced transient GLS1 expression that peaked at 24 h and declined over the following days, consistent with the nature of the mRNA lacking genomic integration (Figure 5O).

Collectively, these results confirmed that mGls@HEV‐MTP met all requirements for precision therapy in IBIs, including hierarchically selective delivery toward infection sites through MTP recognition, acidic microenvironment‐responsive payload release, subsequent macrophage‐selective delivery, and efficient mRNA translation.

### 2.6 Glutaminolysis‐Fueled Anaplerosis Restoration Guided by mGls@HEV‐MTP Reawakens Potent Macrophage Antibacterial Immunity

**FIGURE 6:**
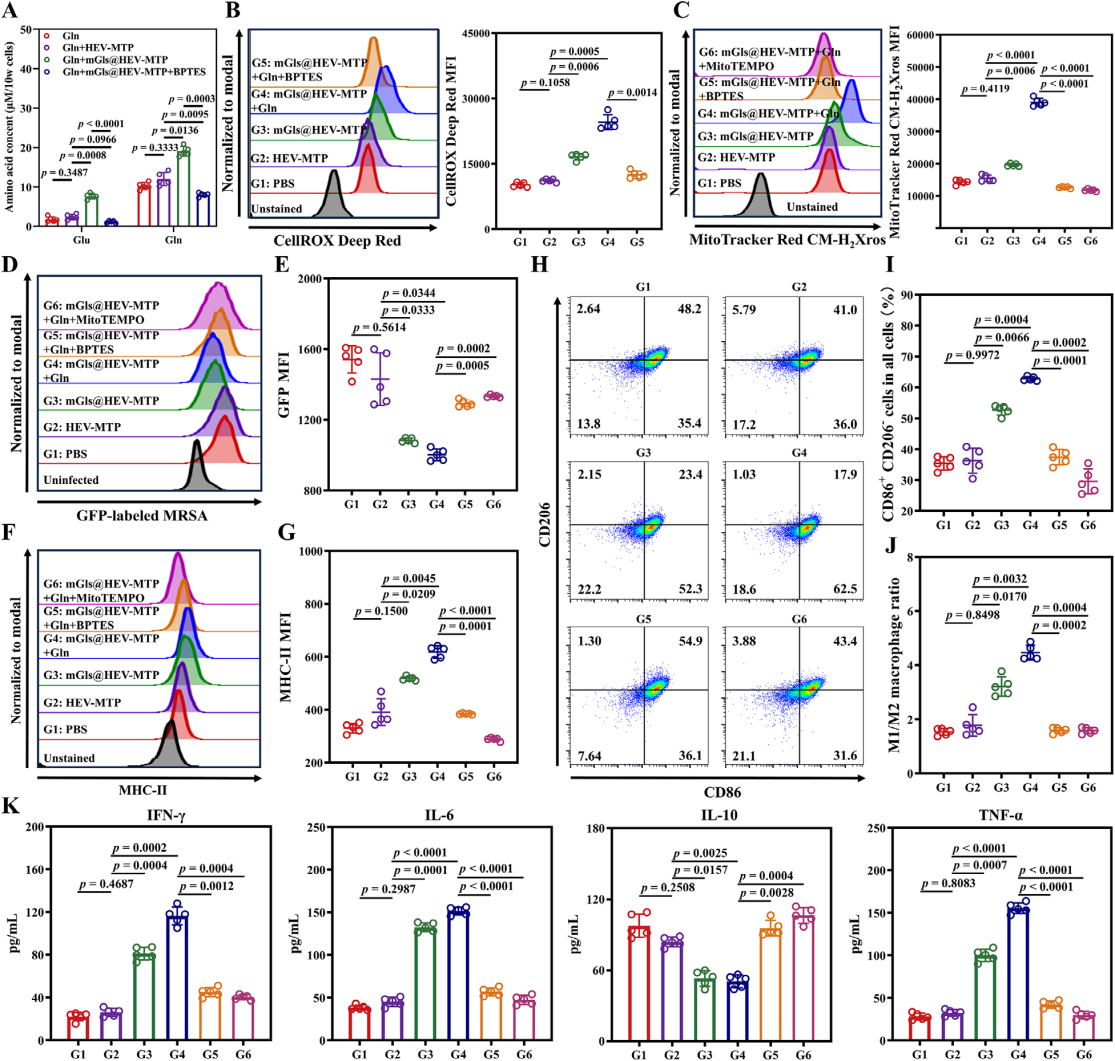
Glutaminolysis‐fueled anaplerosis restoration guided by mGls@HEV‐MTP reawakens potent macrophage antibacterial immunity. (A) The intracellular content of Gln and Glu in infected macrophages after different treatments at 7dpi (*n =* 5). (B) Representative flow cytometry plots and quantification of CellROX Deep Red staining showing intracellular ROS levels of infected macrophages upon different treatments at 7dpi (*n =* 5). (C) Representative flow cytometry plots and quantification of MitoTracker Red CM‐H_2_Xros staining showing intracellular mtROS levels in infected macrophages after different treatments at 7dpi (*n =* 5). (D) Representative flow cytometry plots and (E) quantification of intracellular residual *S. aureus* in differentially treated macrophages at 7dpi (*n =* 5). (F–J) Representative flow cytometry plots and quantification of MHC‐II^+^ cells, CD86^+^ CD206^−^ cells, the ratio of proinflammatory (CD86^+^) to immunosuppressive (CD206^+^) phenotypes in differentially treated macrophages at 7dpi (*n =* 5). (K) The levels of IFN‐γ, IL‐6, IL‐10 and TNF‐α in differentially treated macrophages (*n =* 5). All *n* values indicated the number of independent biological replicates. All data were presented as the mean ± standard deviation. The *p*‐values were determined by (A–K) one‐way ANOVA with Tukey's post hoc test for comparisons among multiple groups.

The antibacterial immune function of macrophages has been reported to highly depend on the functional integrity of glutaminolysis‐fueled anaplerosis in the infection niche [31, 49, 50]. To probe whether mGls@HEV‐MTP‐induced GLS1 overexpression could reawaken macrophage antibacterial function by restoring glutaminolysis‐fueled anaplerosis and promoting mtROS burst, we performed rescue and blockade experiments using mGls@HEV‐MTP in the presence or absence of glutamine, the selective glutaminase inhibitor BPTES, and the mtROS scavenger MitoTEMPO, all of which have been widely used in related studies (Table S6). First, we established infected macrophages by co‐culturing macrophages with *S. aureus* for 2 h, followed by different treatments for 7 days. The mGls@HEV‐MTP was able to increase the intracellular content of Glu and Gln in cells to some extent, whereas this was reversed by BPTES (Figure 6A). As previous studies reported, the simultaneous increase in intracellular Gln and Glu reflects the enhancement of immunometabolic activity during the recovery of macrophage immunocompetence, which does not contradict enhanced glutaminolysis activity [50, 51]. Proinflammatory macrophages are believed to exhibit excellent bactericidal activity, which contributes to the activation of adaptive immune responses [52]. Glutamine, serving as a major carbon source for the TCA cycle in infected macrophages, has been reported to contribute to the metabolic program of the bactericidal macrophage phenotype [31, 50, 53]. In response, we attempted to evaluate whether mGls@HEV‐MTP was capable of reprogramming macrophages towards a bactericidal phenotype. At 7dpi, infected macrophages exhibited no obvious ROS burst after treatment with HEV‐MTP (loading with a noncoding scrambled mRNA control), whereas we observed a significant upregulation of intracellular total ROS levels in the mGls@HEV‐MTP group, further amplified by exogenous glutamine supplementation. However, the total ROS generation was inhibited to a great extent when glutaminolysis of infected macrophages was disturbed, confirming that ROS generation was fueled by glutaminolysis under these conditions (Figure 6B). As demonstrated by the flow cytometry data (Figure 6C–E), mGls@HEV‐MTP, particularly in combination with glutamine supplementation, increased cellular mtROS levels along with elimination of approximately half of intracellular bacteria at 7dpi, confirming that mGls@HEV‐MTP effectively restored the bactericidal function of glutaminolysis‐impaired macrophages. Significantly upregulated mtROS levels in G4 might be attributed not only to enhanced glutaminolysis‐fueled anaplerosis but also to more glutamine available for TCA cycle in macrophages. In parallel, the proportions of MHC‐II^+^ and CD86^+^CD206^−^ macrophages increased after treatment with mGls@HEV‐MTP and glutamine supplementation (Figure 6F–J and Figure S23). Consistent with this phenotypic transition, mGls@HEV‐MTP, particularly in combination with glutamine supplementation, profoundly remodeled the cytokine secretion profile (Figure 6K). Glutaminolysis‐fueled anaplerosis restoration increased IFN‐γ, IL‐6, and TNF‐α production and decreased IL‐10 secretion. However, the mtROS levels and the proportion of bactericidal macrophages sharply decreased when GLS1 was inhibited by BPTES, indicating that metabolic flux through glutaminolysis was required for macrophage immune activation and mtROS production. Notably, mtROS scavenging by MitoTEMPO abrogated these beneficial effects induced by glutaminolysis‐fueled anaplerosis restoration, indicating that mtROS was a necessary downstream mediator of GLS1 overexpression‐driven macrophage antibacterial immunity.

NADPH oxidase 2 (NOX2)‐mediated ROS production is a canonical and widely recognized mechanism for the phagosomal oxidative burst, but it is not the only source of antimicrobial ROS in macrophages [54, 55]. Moreover, mtROS constitutes an additional metabolically regulated antibacterial ROS source [33]. To adequately distinguish this canonical NOX2‐dependent ROS from the metabolically regulated mtROS associated with GLS1‐dependent glutaminolysis, we performed functional inhibition experiments using the NOX2 inhibitor GSK2795039. As aforementioned, MitoTEMPO markedly reduced the mGls@HEV‐MTP‐induced mtROS signal and significantly abrogated the beneficial effects of GLS1 restoration on intracellular bacterial clearance and bactericidal macrophage activation. By contrast, NOX2 inhibition partially impaired intracellular bacterial clearance and total oxidative burst, but did not eliminate the GLS1‐dependent increase in mtROS (Figure S24), which was consistent with the canonical role of NOX2 in antibacterial defense. These results indicate that NOX2 contributes to canonical phagosomal ROS‐dependent bacterial killing, whereas the glutaminolysis‐dependent rescue observed in our system is mediated predominantly through mtROS. Our data do not exclude the contribution of NOX2 to macrophage antibacterial activity. Rather, our results indicate that prolonged infection disrupts a glutaminolysis‐dependent mtROS program, and GLS1 restoration reactivates this mtROS‐dependent arm of macrophage antibacterial immunity. Glutaminolysis mainly controls the mtROS arm of macrophage antibacterial immunity, whereas NOX2 represents a canonical phagosomal ROS pathway that may operate in parallel or cooperate with mtROS.

In conclusion, our data demonstrated that mGls@HEV‐MTP‐mediated Gls mRNA delivery served as an effective tool to reprogram persistently infected macrophages back toward a bactericidal phenotype by enhancing glutaminolysis‐fueled anaplerosis, thereby promoting the elimination of *S. aureus* and subsequent activation of adaptive immune responses.

### 2.7 Therapeutic Efficacy and Immune Landscapes of mGls@HEV‐MTP Against IBIs In Vivo

**FIGURE 7:**
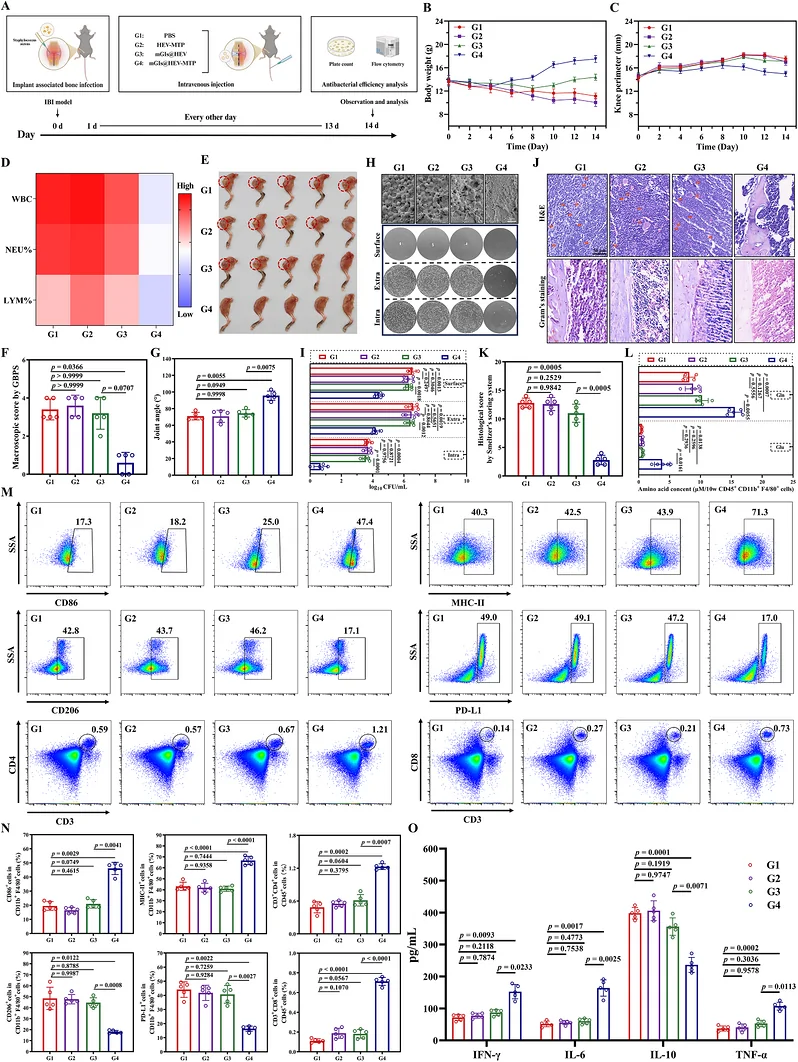
Therapeutic efficacy and immune landscapes of mGls@HEV‐MTP against IBIs in vivo. (A) Schematic timeline for assessing the therapeutic efficacy of mGls@HEV‐MTP in the MRSA‐infected IBI model. This figure was created by BioRender.com. (B) The time curves of body weight and (C) knee perimeter of differentially treated IBI model mice (*n =* 6). (D) Routine blood test results from differentially treated IBI model mice at 14dpi. (E) Photographs of knee joints from differentially treated IBI model mice at 14dpi (*n =* 5). The red dashed box indicates the abscess formation. (F) Corresponding macroscopic scores of knee joints by GBPS and (G) knee flexion angles in differentially treated IBI model mice at 14dpi (*n =* 5). (H) Representative SEM images of *S. aureus* biofilm on the surfaces of titanium rod implants and (I) quantification of cultivation plates of the bone marrow aspirate from differentially treated mice at 14dpi (*n =* 5). Scale bar, 2 µm. (J) Representative images of H&E and Gram's staining, and (K) corresponding histological scores of the tibiae from the differentially treated mice reflecting the severity of the infection at 14dpi (*n =* 5). Arrows indicate neutrophil infiltration. Scale bar, 50 µm. (L) The contents of glutamine and glutamate in peri‐implant macrophages (CD45^+^ CD11b^+^ F4/80^+^ cells) sorted out from peri‐implant bone marrow of IBI model mice by FACS in different groups (*n =* 5). (M) Representative flow cytometry plots of immune cell populations in peri‐implant bone marrow from the tibiae of differentially treated IBI mice, including CD86, MHC‐II, CD206 and PD‐L1 expression levels of macrophages by gating on CD45^+^ CD11b^+^ F4/80^+^ cells and CD4^+^/CD8^+^ T cells by gating on CD45^+^ CD3^+^ cells. (N) Quantification of corresponding immune cell populations in different groups (*n =* 5). (O) The levels of IFN‐γ, IL‐6, IL‐10, and TNF‐α in peri‐implant bone marrow aspirates from the tibiae of differentially treated IBI model mice (*n =* 5). All *n* values indicated the number of independent biological replicates. All data were presented as the mean ± standard deviation. The *p*‐values were determined by (F) the Kruskal–Wallis test with Dunn's multiple‐comparison test and (G,I,K,L,N,O) one‐way ANOVA with Tukey's post hoc test for comparisons among multiple groups.

Having confirmed the feasibility of glutaminolysis‐fueled anaplerosis restoration in vitro, we next evaluated whether this strategy could achieve meaningful antibacterial effects in vivo. Considering that nearly half of *S. aureus* clinical isolates from IBIs are MRSA, a representative MRSA isolate (USA300) was chosen to establish the in vivo IBI model [2]. The IBI model mice received the intravenous injection of PBS (G1, 5 mL/kg), HEV‐MTP (G2, 5 mg protein/kg, loading with a noncoding scrambled mRNA control), mGls@HEV (G3, 5 mg protein/kg) and mGls@HEV‐MTP (G4, 5 mg protein/kg) from 1dpi every other day until 14dpi (Figure 7A). Since the administration route used in this study was intravenous injection, it remained critically important to evaluate the systemic toxicity of the nanovesicles. These mice were euthanized at 14dpi, and the major organs were collected for histological analysis using hematoxylin and eosin (H&E) staining. No obvious tissue damage was observed in the major organs of the nanovesicle‐injected mice compared to the PBS‐injected mice (Figure S25). Besides, we harvested the knee joints from IBI model mice receiving different treatments at 14dpi to further evaluate the therapeutic efficacy of mGls@HEV‐MTP in prolonged IBIs.

Compared with the other groups, mice treated with mGls@HEV‐MTP (G4) exhibited markedly reduced infection severity, as evidenced by alleviated knee joint swelling and the recovery of body weight (Figure 7B,C). Additionally, the pronounced decrease in the percentage of neutrophils (NEU%) of IBI model mice receiving mGls@HEV‐MTP indicated a relieved systemic inflammatory response (Figure 7D). Because of immune compromise in prolonged infection, substantial proliferation of *S. aureus* led to the formation of a massive abscess within the joint (Figure 7E). Notably, mGls@HEV‐MTP markedly attenuated knee joint abscesses and reduced pathological scores (Figure 7F). Orthopedic IBIs often require a treatment strategy that extends beyond bacterial eradication to preserve joint function. Interestingly, prolonged infection reduced the joint flexion angle (Figure 7G), indicating impaired mobility, whereas mGls@HEV‐MTP treatment effectively restored joint flexibility, outperforming other treatment groups. Then, the titanium rod implants and bone marrow aspirates were collected from differentially treated mice for further experiments. The scanning electron microscopy (SEM) images revealed near‐complete biofilm disruption on the implants in G4, with only deformed bacterial remnants visible (Figure 7H). Correspondingly, the plate‐counting results confirmed that mGls@HEV‐MTP achieved significant bacterial clearance efficiencies of approximately 98.7% for peri‐implant bacteria and 99.7% intracellular bacteria in MRSA‐infected tibias (Figure 7I). Moreover, histopathological analysis showed reduced neutrophil infiltration and bacteria‐positive regions as well as histological scores in G4, suggesting that mGls@HEV‐MTP‐mediated immunotherapy achieved substantial pathological remission (Figure 7J,K). To confirm the glutaminolysis‐fueled anaplerosis restoration mediated by mGls@HEV‐MTP in vivo, we isolated macrophages from peri‐implant bone marrow of IBI model mice. The intracellular levels of glutamine and glutamate were both significantly upregulated in G4, further supporting that mGls@HEV‐MTP was able to reprogram glutamine metabolism in peri‐implant macrophages in defense against MRSA infection in vivo (Figure 7L).

The functional integrity of macrophages acts as a pivotal bridge between innate and adaptive immunity, and is essential for comprehensive immune system activation. Following pathogen phagocytosis, macrophages degrade bacterial antigens into small peptide fragments to form antigen‐MHC‐II complexes. Subsequently, the antigen‐MHC‐II complexes are translocated to the cell surface for antigen presentation, which directly primes CD4^+^ T cells and initiates antigen‐specific humoral and cellular immune responses [56]. However, in the chronic infection context characterized by biofilm formation and intracellular bacterial persistence, macrophages were skewed into an immunocompromised state, which was featured by macrophages with an anti‐inflammatory phenotype. Notably, immune profiling of infected peri‐implant bone marrow demonstrated that mGls@HEV‐MTP activated multiple immune populations involved in antibacterial immunity (Figure 7M,N). The mGls@HEV‐MTP treatment significantly elevated the fraction of proinflammatory macrophages, as evidenced by the increased CD86 and reduced CD206 expression. Similarly, these mice receiving mGls@HEV‐MTP exhibited a nearly 2.0‐fold increase in the proportion of MHC‐II^+^ macrophages in peri‐implant bone marrow, indicating an enhanced ability of bacterial engulfment and antigen presentation. Crucially, the downregulation of PD‐L1 in peri‐implant macrophages further verified the transition from an immunosuppressive state to an immune‐reawakening state following the treatment with mGls@HEV‐MTP. The higher infiltration of CD4^+^ T cells represented stronger humoral immunity [57]. Compared with other groups, significantly higher levels of CD4^+^ T cell (CD45^+^ CD3^+^ CD4^+^) infiltration were observed at the infection sites after treatment with mGls@HEV‐MTP, suggesting an activated adaptive immune response. Given that intracellular sequestration was a sophisticated immune evasion strategy adopted by invading pathogens, CD8^+^ T cells were essential for identifying and eliminating infected cells [58]. Notably, the mGls@HEV‐MTP treatment promoted the robust recruitment of CD8^+^ T cells (CD45^+^ CD3^+^ CD8^+^) to the infectious sites, facilitating the effective clearance of intracellular bacteria. To further elucidate the immunomodulatory effects of mGls@HEV‐MTP, we analyzed the cytokine profiles in the infection microenvironment. The mice receiving mGls@HEV‐MTP showed a pronounced increase in proinflammatory cytokines such as IL‐6, TNF‐α, and IFN‐γ and a decrease in anti‐inflammatory cytokines such as IL‐10 (Figure 7O), further evidencing that mGls@HEV‐MTP was capable of eliciting robust antibacterial immune responses in the typically immunosuppressive microenvironment of prolonged IBIs.

Together, these results suggested that the eradication of IBIs was not solely attributable to mRNA‐mediated immune activation. Crucially, by efficiently targeting *S. aureus*, mGls@HEV‐MTP endowed poorly immunogenic *S. aureus* with highly immune‐recognition signals in the chronic infection microenvironment, thereby expediting bacterial immune clearance.

## 3 Discussion

Our work uncovered the pivotal role of glutaminolysis‐fueled anaplerosis in macrophage immunocompetence, providing a promising insight to overcome both immunosuppression and poor immunogenicity in prolonged IBIs. By mapping the landscape of IBI pathological progression at the metabolic and transcriptional levels, we revealed that GLS1 downregulation‐mediated glutaminolysis‐fueled dysfunction functioned as a fundamental determinant of macrophage immune dysfunction in prolonged IBIs. Mechanistically, glutaminolysis‐fueled dysfunction stifles the mtROS burst required for bactericidal activity and antigen presentation, driving macrophages from a bactericidal phenotype towards an immunologically dysfunctional state. Thus, we engineered a hierarchically selective therapeutic system (mGls@HEV‐MTP) for glutaminolysis‐fueled restoration in macrophages via hierarchical infection‐to‐macrophage selective GLS1 mRNA delivery. Unlike conventional macrophage‐specific systems, our system enables hierarchical infection‐to‐macrophage targeting via the affinity of MTP for *S. aureus* and the subsequent cleavage of hydrazone bond within the acidic infection niche, concomitantly promoting bactericidal macrophage repolarization and marking invading pathogens with immune‐recognition signals, thereby addressing both immunosuppression and poor immunogenicity in the infection niche.

Although our data support glutaminolysis‐fueled anaplerosis restoration as a strategy to reactivate antibacterial macrophage metabolism, GLS1 is a central metabolic enzyme with broad physiological and pathological functions, and its sustained activation may produce pleiotropic effects beyond antibacterial immune reprogramming. In line with this concern, Leone et al. showed that systemic glutamine blockade in tumors induced divergent metabolic programs in different cell types: it suppressed oxidative and glycolytic metabolism in tumor cells, thereby reducing hypoxia, acidosis, and nutrient depletion, whereas effector T cells adapted by enhancing oxidative metabolism and acquiring a highly activated, long‐lived phenotype [59]. This study highlights that the biological consequence of manipulating glutamine metabolism is highly niche‐ and cell type‐dependent.

The same consideration is relevant to macrophage biology. The precise role of glutamine metabolism in dictating macrophage immune function remains a subject of intense debate, presenting a “metabolic paradox” where this amino acid appears to drive divergent immune states. Some studies support glutamine as an indispensable driver of proinflammatory responses [10, 11, 31, 50]. In the infection microenvironment of IBIs, glutaminolysis‐fueled restoration promoted glutamine‐derived anaplerosis, mtROS generation, and macrophage bactericidal polarization. However, contradictory studies highlight a pivotal role of glutamine in sustaining immunosuppressive phenotypes and fostering immune tolerance. In immunosuppressive macrophages, a significant fraction of glutamine‐derived nitrogen is diverted into the hexosamine biosynthetic pathway (HBP) to synthesize UDP‐GlcNAc, a substrate essential for *N*‐glycosylation of lectin receptors such as CD206 [49, 60]. In certain tumor microenvironments, restricting glutamine availability has surprisingly been shown to skew tumor‐associated macrophages (TAMs) from an immunosuppressive state back to a proinflammatory phenotype, suggesting that glutamine can actively enforce immunosuppression [61]. The enhanced glutaminolysis‐fueled anaplerosis may favor antibacterial immunity activation within an infection niche, but may produce different or even opposing macrophage states in repair‐dominant or tumor‐associated microenvironments. This dichotomy suggests that GLS1‐mediated glutaminolysis‐fueled restoration is not intrinsically immune‐activating or immunosuppressive; rather, its functional outcome acts as a niche‐dependent immunometabolic checkpoint. Similarly, mtROS should be viewed as a dose‐ and duration‐dependent mediator. Controlled mtROS production contributes to macrophage antimicrobial defense and bacterial killing, but excessive or prolonged mtROS may amplify oxidative tissue damage, inflammatory injury, and delayed resolution [62]. These issues are especially relevant for IBIs, where antibacterial immune activation must be balanced with tissue repair. Sustained inflammation and oxidative burst may facilitate bacterial clearance, but they could also impair bone regeneration or exacerbate peri‐implant tissue damage after infection control. Thus, therapeutic GLS1 restoration should aim to normalize defective mtROS generation during chronic infection rather than induce persistent oxidative activation.

Another important translational concern is the potential risk of ectopic or sustained GLS1 overexpression. GLS1 is frequently exploited by proliferating cells and cancer cells to support glutamine addiction, anabolic metabolism, redox balance, invasion, and metastasis [63, 64]. Although our hierarchically selective therapeutic system is designed for macrophage‐directed modulation at infection sites, unexpected GLS1 expression in non‐target cells, such as endothelial cells and occult tumor cells, could theoretically cause undesirable effects. In addition, GLS1 has essential physiological roles in non‐target organs, particularly in the central nervous system and the kidney, in which overactive glutamine metabolism may lead to neuroinflammation, synaptic dysfunction, complement‐associated tubulointerstitial injury, and renal fibrosis [65, 66].

Accordingly, the optimal translational strategy should not involve sustained systemic GLS1 activation, but rather spatiotemporally restricted and macrophage‐selective GLS1 modulation. Future studies should define the minimal effective dose, duration of GLS1 expression in vivo, reversibility of metabolic reprogramming, oxidative damage, bone repair, tumor‐promoting potential, and long‐term safety after repeated administration.

## 4 Experimental Section

Detailed experimental procedures are provided in the Supplementary Materials.

## Author Contributions

W.N.Y. conceived and designed the project, conducted and analyzed experiments, interpreted the data, and wrote the manuscript. Z.H.A. plotted part of the schematic diagrams in this paper, facilitated the progression of the project, assisted with interpretation of the data and analyzed experiments. H.L.C. analyzed the bulk RNA‐seq and single‐cell RNA‐seq data. C.S.L. and J.X.Z. helped with cell and animal experiments. W.S.C., W.T.C., and Z.Z.S. designed mitochondrial experiments and assisted with interpretation of data. J.X., T.S., T.Z. and S.C.W. helped with bacterial experiments. M.C. and Z.R.G. assisted with the design and establishment of the nanotherapeutic system. J.B.X., A.L. and H.B.W. critically appraised the manuscript and provided research funding support.

## Funding

This work was supported by the National Natural Science Foundation of China (Grant No. 82372487, 32371412, 32071349, 82201730), Key research and development program of Zhejiang Province (Grant No.2025C02167), the 5510 Project of the Second Affiliated Hospital, Zhejiang University School of Medicine (Grant No. 551005322022), Natural Science Foundation of Zhejiang province, China (Grant No. LY24C100001, LQ22H060004), the Zhejiang Provincial Medical Science and Technology Project of China (Grant No. 2023KY111), Central Guidance on Local Science and Technology Development Fund of Zhejiang Province (Grant No. 2024ZY01033), Zhejiang Province Traditional Chinese Medicine Science and Technology Project (Grant No. 2023ZL491), and the Research Project on Industry‐Education Integration Innovation for New Quality Productive Forces organized by the Research Project on Industry‐Education Integration Innovation for New Quality Productive Forces organized by the Center for School Development and Planning, Ministry of Education (Grant No. CSDP26LF8A408).

## Ethics Statement

This study complied with the ARRIVE 2.0 reporting guidelines for in vivo animal research and received approval from the Animal Ethics Committee of The Second Affiliated Hospital, Zhejiang University School of Medicine (SAHZU‐2024‐240). Moreover, this study was conducted in accordance with the ethical principles of the Declaration of Helsinki, following approval by the Ethics Committee of Human Research of the Second Affiliated Hospital, Zhejiang University School of Medicine (SAHZU‐2024‐1218). Written informed consent was obtained from all participants prior to sample acquisition.

## Conflicts of Interest

The authors declare no conflicts of interest.

## Supporting information




**Supporting File**: advs77500‐sup‐0001‐SuppMat.docx.

## Data Availability

The data that supports the findings of this study are available in the supplementary material of this article.

## References

[1] E. A. Masters , B. F. Ricciardi , B. KLdM , T. F. Moriarty , E. M. Schwarz , and G. Muthukrishnan , “Skeletal Infections: Microbial Pathogenesis, Immunity and Clinical Management,” Nature Reviews Microbiology 20, no. 7 (2022): 385–400. 35169289 10.1038/s41579-022-00686-0 PMC8852989

[2] C. R. Arciola , D. Campoccia , and L. Montanaro , “Implant Infections: Adhesion, Biofilm Formation and Immune Evasion,” Nature Reviews Microbiology 16, no. 7 (2018): 397–409. 29720707 10.1038/s41579-018-0019-y

[3] F. Peyrusson , H. Varet , T. K. Nguyen , et al., “Intracellular Staphylococcus Aureus Persisters Upon Antibiotic Exposure,” Nature Communications 11, no. 1 (2020): 2200. 10.1038/s41467-020-15966-7 PMC7198484 32366839

[4] M. Casanova‐Acebes , E. Dalla , A. M. Leader , et al., “Tissue‐Resident Macrophages Provide a Pro‐Tumorigenic Niche To Early Nsclc Cells,” Nature 595, no. 7868 (2021): 578–584. 34135508 10.1038/s41586-021-03651-8 PMC8923521

[5] M. J. Sweet , D. Ramnath , A. Singhal , and R. Kapetanovic , “Inducible Antibacterial Responses in Macrophages,” Nature Reviews Immunology 25, no. 2 (2025): 92–107. 10.1038/s41577-024-01080-y 39294278

[6] H.‐L. Yang , Z.‐Z. Lai , J.‐W. Shi , et al., “A Defective Lysophosphatidic Acid‐Autophagy Axis Increases Miscarriage Risk By Restricting Decidual Macrophage Residence,” Autophagy 18, no. 10 (2022): 2459–2480. 35220880 10.1080/15548627.2022.2039000 PMC9542369

[7] D. A. Mogilenko , A. Sergushichev , and M. N. Artyomov , “Systems Immunology Approaches to Metabolism,” Annual Review of Immunology 41, no. 1 (2023): 317–342. 10.1146/annurev-immunol-101220-031513 37126419

[8] A. E. Ogunware and T. Kielian , “Immunometabolism Shapes Chronic Staphylococcus Aureus Infection: Insights From Biofilm Infection Models,” Current Opinion in Microbiology 86 (2025): 102612. 40409167 10.1016/j.mib.2025.102612 PMC12276999

[9] Z. Van Roy , P. Arumugam , B. P. Bertrand , D. D. Shinde , V. C. Thomas , and T. Kielian , “Tissue Niche Influences Immune and Metabolic Profiles to Staphylococcus Aureus Biofilm Infection,” Nature Communications 15, no. 1 (2024): 8965. 10.1038/s41467-024-53353-8 PMC11487069 39420209

[10] P.‐S. Liu , Y.‐T. Chen , X. Li , et al., “CD40 Signal Rewires Fatty Acid and Glutamine Metabolism for Stimulating Macrophage Anti‐Tumorigenic Functions,” Nature Immunology 24, no. 3 (2023): 452–462. 36823405 10.1038/s41590-023-01430-3 PMC9977680

[11] R. J. W. Arts , B. Novakovic , R. ter Horst , et al., “Glutaminolysis and Fumarate Accumulation Integrate Immunometabolic and Epigenetic Programs in Trained Immunity,” Cell Metabolism 24, no. 6 (2016): 807–819. 27866838 10.1016/j.cmet.2016.10.008 PMC5742541

[12] S. Qin , X. Tang , Y. Chen , et al., “mRNA‐based therapeutics: Powerful and Versatile Tools to Combat Diseases,” Signal Transduction and Targeted Therapy 7, no. 1 (2022): 166. 35597779 10.1038/s41392-022-01007-w PMC9123296

[13] E. Kon , N. Ad‐El , I. Hazan‐Halevy , L. Stotsky‐Oterin , and D. Peer , “Targeting Cancer with mRNA–lipid Nanoparticles: Key Considerations and Future Prospects,” Nature Reviews Clinical Oncology 20, no. 11 (2023): 739–754. 10.1038/s41571-023-00811-9 37587254

[14] X. Hou , T. Zaks , R. Langer , and Y. Dong , “Lipid Nanoparticles for mRNA Delivery,” Nature Reviews Materials 6, no. 12 (2021): 1078–1094. 34394960 10.1038/s41578-021-00358-0 PMC8353930

[15] C. Schwechheimer and M. J. Kuehn , “Outer‐Membrane Vesicles from Gram‐Negative Bacteria: Biogenesis And Functions,” Nature Reviews Microbiology 13, no. 10 (2015): 605–619. 26373371 10.1038/nrmicro3525 PMC5308417

[16] M. Li , H. Zhou , C. Yang , et al., “Bacterial Outer Membrane Vesicles as a Platform for Biomedical Applications: An Update,” Journal of Controlled Release 323 (2020): 253–268. 32333919 10.1016/j.jconrel.2020.04.031

[17] A. Urso , I. R. Monk , Y.‐T. Cheng , et al., “Staphylococcus Aureus Adapts to Exploit Collagen‐Derived Proline During Chronic Infection,” Nature Microbiology 9, no. 10 (2024): 2506–2521. 10.1038/s41564-024-01769-9 PMC11445067 39134708

[18] J. A. Freiberg , V. M. Reyes Ruiz , B. D. Gimza , et al., “Restriction of Arginine Induces Antibiotic Tolerance in Staphylococcus Aureus,” Nature Communications 15, no. 1 (2024): 6734. 10.1038/s41467-024-51144-9 PMC11306626 39112491

[19] A. Aalirezaie , T. W. Bauer , H. Fayaz , W. Griffin , et al., “Hip and Knee Section, Diagnosis, Reimplantation: Proceedings of International Consensus on Orthopedic Infections,” The Journal of Arthroplasty 34, (2019): S369–S379. 30343965 10.1016/j.arth.2018.09.021

[20] E. J. McPherson , C. Woodson , P. Holtom , N. Roidis , C. Shufelt , and M. Patzakis , “Periprosthetic Total Hip Infection,” Clinical Orthopaedics and Related Research 403 (2002): 8–15. 12360001

[21] A.‐P. Puhto , T. M. Puhto , T. T. Niinimäki , J. I. Leppilahti , and H. P. T. Syrjälä , “Two‐Stage Revision for Prosthetic Joint Infection: Outcome and Role of Reimplantation Microbiology in 107 Cases,” The Journal of Arthroplasty 29, no. 6 (2014): 1101–1104. 24461248 10.1016/j.arth.2013.12.027

[22] M. B. Ka , A. Daumas , J. Textoris , and J.‐L. Mege , “Phenotypic Diversity and Emerging New Tools to Study Macrophage Activation in Bacterial Infectious Diseases,” Frontiers in Immunology 5 (2014): 500. 25346736 10.3389/fimmu.2014.00500 PMC4193331

[23] Q. Peng , X. Qiu , Z. Zhang , et al., “PD‐L1 on Dendritic Cells Attenuates T cell Activation and Regulates Response to Immune Checkpoint Blockade,” Nature Communications 11, no. 1 (2020): 4835. 10.1038/s41467-020-18570-x PMC7518441 32973173

[24] X. Sun , J. Wu , L. Liu , et al., “Transcriptional Switch of Hepatocytes Initiates Macrophage Recruitment and T‐cell Suppression in Endotoxemia,” Journal of Hepatology 77, no. 2 (2022): 436–452. 35276271 10.1016/j.jhep.2022.02.028

[25] S. Aibar , C. B. González‐Blas , T. Moerman , et al., “SCENIC: Single‐Cell Regulatory Network Inference and Clustering,” Nature Methods 14, no. 11 (2017): 1083–1086. 28991892 10.1038/nmeth.4463 PMC5937676

[26] K. J. Yamada , C. E. Heim , X. Xi , et al., “Monocyte Metabolic Reprogramming Promotes Pro‐Inflammatory Activity and Staphylococcus Aureus Biofilm Clearance,” PLOS Pathogens 16, no. 3 (2020): 1008354. 10.1371/journal.ppat.1008354 PMC7080272 32142554

[27] L. A. J. O'Neill and R. J. Kishton , “A Guide to Immunometabolism for Immunologists,” Nature Reviews Immunology 16, no. 9 (2016): 553–565. 10.1038/nri.2016.70 PMC5001910 27396447

[28] L. Yang , Z. Chu , M. Liu , et al., “Amino Acid Metabolism in Immune Cells: Essential Regulators of the Effector Functions, and Promising Opportunities to Enhance Cancer Immunotherapy,” Journal of Hematology & Oncology 16, no. 1 (2023): 59. 37277776 10.1186/s13045-023-01453-1 PMC10240810

[29] O. Goldmann and E. Medina , “Myeloid‐Derived Suppressor Cells Impair CD4+ T cell Responses During Chronic Staphylococcus Aureus Infection Via Lactate Metabolism,” Cellular and Molecular Life Sciences 80, no. 8 (2023): 221. 37480485 10.1007/s00018-023-04875-9 PMC10363054

[30] C. E. Heim , M. E. Bosch , K. J. Yamada , et al., “Lactate Production by Staphylococcus Aureus Biofilm Inhibits HDAC11 to Reprogramme the Host Immune Response During Persistent Infection,” Nature Microbiology 5, no. 10 (2020): 1271–1284. 10.1038/s41564-020-0756-3 PMC7529909 32661313

[31] L. C. Davies , C. M. Rice , E. M. Palmieri , P. R. Taylor , D. B. Kuhns , and D. W. McVicar , “Peritoneal Tissue‐Resident Macrophages are Metabolically Poised to Engage Microbes Using Tissue‐Niche Fuels,” Nature Communications 8, no. 1 (2017): 2074. 10.1038/s41467-017-02092-0 PMC5727035 29234000

[32] F. J. Roca , L. J. Whitworth , H. A. Prag , M. P. Murphy , and L. Ramakrishnan , “Tumor Necrosis Factor Induces Pathogenic Mitochondrial ROS in Tuberculosis Through Reverse Electron Transport,” Science 376, no. 6600 (2022): abh2841. 10.1126/science.abh2841 PMC7612974 35737799

[33] A. P. West , I. E. Brodsky , C. Rahner , et al., “TLR Signalling Augments Macrophage Bactericidal Activity Through Mitochondrial ROS,” Nature 472, no. 7344 (2011): 476–480. 21525932 10.1038/nature09973 PMC3460538

[34] J. Garaude , R. Acín‐Pérez , S. Martínez‐Cano , et al., “Mitochondrial Respiratory‐Chain Adaptations in Macrophages Contribute to Antibacterial Host Defense,” Nature Immunology 17, no. 9 (2016): 1037–1045. 27348412 10.1038/ni.3509 PMC4994870

[35] E. L. Mills , B. Kelly , A. Logan , et al., “Succinate Dehydrogenase Supports Metabolic Repurposing of Mitochondria to Drive Inflammatory Macrophages,” Cell 167, no. 2 (2016): 457–470.e13. 27667687 10.1016/j.cell.2016.08.064 PMC5863951

[36] B. H. Abuaita , T. L. Schultz , and M. X. O'Riordan , “Mitochondria‐Derived Vesicles Deliver Antimicrobial Reactive Oxygen Species to Control Phagosome‐Localized Staphylococcus aureus,” Cell Host & Microbe 24, no. 5 (2018): 625–636.e5. 30449314 10.1016/j.chom.2018.10.005 PMC7323595

[37] A. Fontalis , A. T. Yasen , D. E. Giebaly , T. D. Luo , A. Magan , and F. S. Haddad , “Optimizing Debridement and Implant Retention in Acute Periprosthetic Joint Infections,” The Bone & Joint Journal 106‐B, no. 12 (2024): 1377–1384. 10.1302/0301-620X.106B12.BJJ-2024-0282.R1 39615530

[38] F. Chen , Y. Xue , X. Ma , et al., “Hierarchical Targeting of TREM2 + Myeloid Cells via Acid‐Triggered OMVs Reprogram Immunosuppression and Suppress Osteolysis in Bone‐Metastatic TNBC,” Advanced Science 13, no. 32 (2026): 17369. 10.1002/advs.202517369 PMC13252654 41945876

[39] Q. Liu , Y. Li , X. Zhao , X. Yang , Q. Liu , and Q. Kong , “Construction of Escherichia coli Mutant with Decreased Endotoxic Activity by Modifying Lipid A Structure,” Marine Drugs 13, no. 6 (2015): 3388–3406. 26023843 10.3390/md13063388 PMC4483635

[40] O. Y. Kim , H. T. Park , N. T. H. Dinh , et al., “Bacterial Outer Membrane Vesicles Suppress tumor by Interferon‐γ‐mediated Antitumor Response,” Nature Communications 8, no. 1 (2017): 626. 10.1038/s41467-017-00729-8 PMC5606984 28931823

[41] S. R. Paludan , T. Pradeu , S. L. Masters , and T. H. Mogensen , “Constitutive Immune Mechanisms: Mediators of Host Defence and Immune Regulation,” Nature Reviews Immunology 21, no. 3 (2020): 137–150. 10.1038/s41577-020-0391-5 PMC7418297 32782357

[42] S. Carpenter and L. A. J. O'Neill , “From Periphery to Center Stage: 50 years of Advancements in Innate Immunity,” Cell 187, no. 9 (2024): 2030–2051. 38670064 10.1016/j.cell.2024.03.036 PMC11060700

[43] H.‐C. Flemming , J. Wingender , U. Szewzyk , P. Steinberg , S. A. Rice , and S. Kjelleberg , “Biofilms: An emergent form of Bacterial Life,” Nature Reviews Microbiology 14, no. 9 (2016): 563–575. 27510863 10.1038/nrmicro.2016.94

[44] Y. Fan , X.‐D. Li , P.‐P. He , et al., “A Biomimetic Peptide Recognizes and Traps Bacteria In Vivo as Human Defensin‐6,” Science Advances 6, no. 19 (2020): aaz4767. 10.1126/sciadv.aaz4767 PMC7209993 32494712

[45] T. Yang , Y. Mochida , X. Liu , et al., “Conjugation of Glucosylated Polymer Chains to Checkpoint Blockade Antibodies Augments their Efficacy and Specificity for Glioblastoma,” Nature Biomedical Engineering 5, no. 11 (2021): 1274–1287. 10.1038/s41551-021-00803-z 34635819

[46] M. Yao , L. Chen , X. Jin , et al., “Bacteriophage Immunotherapy with Artificial Antigen‐Directed Immune Labeling Against Bacterial Infection,” Science Advances 11, no. 35 (2025): adr1911. 10.1126/sciadv.adr1911 PMC12396327 40880477

[47] G. Li , Z. Feng , Z. Hou , et al., “Dual‐Cascade Responsive H_2_Se Delivery Nanogels Bearing Selenobenzamide as H_2_Se Donor for the Treatment of Multidrug Resistant Bacteria Infections,” Advanced Functional Materials 35, no. 19 (2025): 2418229.

[48] A. J. Grippin , C. Marconi , S. Copling , et al., “SARS‐CoV‐2 mRNA Vaccines Sensitize Tumours to Immune Checkpoint Blockade,” Nature 647, no. 8089 (2025): 488–497. 41125896 10.1038/s41586-025-09655-y PMC12611756

[49] A. K. Jha , S. C.‐C. Huang , A. Sergushichev , et al., “Network Integration of Parallel Metabolic and Transcriptional Data Reveals Metabolic Modules that Regulate Macrophage Polarization,” Immunity 42, no. 3 (2015): 419–430. 25786174 10.1016/j.immuni.2015.02.005

[50] Q. Jiang , Y. Qiu , I. J. Kurland , et al., “Glutamine Is Required for M1‐like Polarization of Macrophages in Response to Mycobacterium tuberculosis Infection,” MBio 13, no. 4 (2022): 0127422. 10.1128/mbio.01274-22 PMC9426538 35762591

[51] F. Fei , K. M. Lee , B. E. McCarry , and D. M. E. Bowdish , “Age‐Associated Metabolic Dysregulation in Bone Marrow‐Derived Macrophages Stimulated with Lipopolysaccharide,” Scientific Reports 6, no. 1 (2016): 22637. 26940652 10.1038/srep22637 PMC4778050

[52] K. B. R. Belchamber , R. Singh , C. M. Batista , et al., “Defective Bacterial Phagocytosis is Associated with Dysfunctional Mitochondria in COPD Macrophages,” European Respiratory Journal 54, no. 4 (2019): 1802244. 31320451 10.1183/13993003.02244-2018

[53] G. M. Tannahill , A. M. Curtis , J. Adamik , et al., “Succinate is an Inflammatory Signal that Induces IL‐1β through HIF‐1α,” Nature 496, no. 7444 (2013): 238–242. 23535595 10.1038/nature11986 PMC4031686

[54] S. B. Berger , X. Romero , C. Ma , et al., “SLAM is a Microbial Sensor that Regulates Bacterial Phagosome Functions in Macrophages,” Nature Immunology 11, no. 10 (2010): 920–927. 20818396 10.1038/ni.1931 PMC3338319

[55] W. Zheng , M. Umitsu , I. Jagan , et al., “An Interaction Between Scribble and the NADPH Oxidase Complex Controls M1 Macrophage Polarization and Function,” Nature Cell Biology 18, no. 11 (2016): 1244–1252. 27694890 10.1038/ncb3413

[56] M. Koyama , P. Mukhopadhyay , I. S. Schuster , et al., “MHC Class II Antigen Presentation by the Intestinal Epithelium Initiates Graft‐versus‐Host Disease and Is Influenced by the Microbiota,” Immunity 51, no. 5 (2019): 885–898.e7. 31542340 10.1016/j.immuni.2019.08.011 PMC6959419

[57] S. L. Mathiasen , L. Gall‐Mas , I. S. Pateras , et al., “Bacterial Genotoxins Induce T‐cell Senescence,” Cell Reports 35, no. 10 (2021): 109220. 34107253 10.1016/j.celrep.2021.109220

[58] T. Torcellan , C. Friedrich , R. Doucet‐Ladevèze , et al., “Circulating NK Cells Establish Tissue Residency Upon Acute Infection of Skin and Mediate Accelerated Effector Responses to Secondary Infection,” Immunity 57, no. 1 (2024): 124–140.e7. 38157853 10.1016/j.immuni.2023.11.018 PMC10783803

[59] R. D. Leone , L. Zhao , J. M. Englert , et al., “Glutamine Blockade Induces Divergent Metabolic Programs to Overcome Tumor Immune Evasion,” Science 366, no. 6468 (2019): 1013–1021. 31699883 10.1126/science.aav2588 PMC7023461

[60] P.‐S. Liu , H. Wang , X. Li , et al., “α‐ketoglutarate Orchestrates Macrophage Activation Through Metabolic and Epigenetic Reprogramming,” Nature Immunology 18, no. 9 (2017): 985–994. 28714978 10.1038/ni.3796

[61] E. M. Palmieri , A. Menga , R. Martín‐Pérez , et al., “Pharmacologic or Genetic Targeting of Glutamine Synthetase Skews Macrophages toward an M1‐like Phenotype and Inhibits Tumor Metastasis,” Cell Reports 20, no. 7 (2017): 1654–1666. 28813676 10.1016/j.celrep.2017.07.054 PMC5575233

[62] F. R. Palma , B. N. Gantner , M. J. Sakiyama , et al., “ROS Production by Mitochondria: Function or Dysfunction?,” Oncogene 43, no. 5 (2023): 295–303. 38081963 10.1038/s41388-023-02907-z

[63] J. Yang , F. Chen , L. Lang , et al., “Therapeutic Targeting of the GLS1–c‐Myc Positive Feedback Loop Suppresses Glutaminolysis and Inhibits Progression of Head and Neck Cancer,” Cancer Research 84, no. 19 (2024): 3223–3234. 39024547 10.1158/0008-5472.CAN-24-0254 PMC11444885

[64] L. Xiang , J. Mou , B. Shao , et al., “Glutaminase 1 Expression in Colorectal Cancer Cells is Induced by Hypoxia and Required for Tumor Growth, Invasion, and Metastatic Colonization,” Cell Death & Disease 10, no. 2 (2019): 40. 30674873 10.1038/s41419-018-1291-5 PMC6426853

[65] H. Chen , S. Fu , X. Li , et al., “Microglial glutaminase 1 Mediates Chronic Restraint Stress‐Induced Depression‐Like Behaviors and Synaptic Damages,” Signal Transduction and Targeted Therapy 8, no. 1 (2023): 452. 38097558 10.1038/s41392-023-01699-8 PMC10721840

[66] Y. Cai , B. Tian , Y. Deng , et al., “Glutamine Metabolism Promotes Renal Fibrosis through Regulation of Mitochondrial Energy Generation and Mitochondrial Fission,” International Journal of Biological Sciences 20, no. 3 (2024): 987–1003. 38250160 10.7150/ijbs.89960 PMC10797689

