# Supplementary material for: Reversing Macrophage Immunometabolic Dysfunction in Prolonged Infections Via a Glutaminolysis‐Fueled Restoration‐Based Hierarchically Selective Therapeutic System
Source: Adv Sci (Weinh). 2026 Sep 10:e77500. Online ahead of print. doi: 10.1002/advs.77500 (PMC13562776; doi:10.1002/advs.77500)
Supplement: Supplementary file 1 [file ADVS-9999-e77500-s001.docx]

***Supplementary Materials for***

**Reversing Macrophage Immunometabolic Dysfunction in Prolonged Infections Via a Glutaminolysis-Fueled Restoration-Based Hierarchically Selective Therapeutic System**

Weinan Yang^*^, Zihao An^*^, Honglu Cai^*^, Congsun Li, Jiaxuan Zou, Wushi Cui, Wentao Chen, Tao Zhang, Shicheng Wang, Zizhan Su, Jian Xiao, Tao Sun, Bin Hu, Min Cheng, Zhongru Gou, Jianbin Xu^#^, An Liu^#^, Haobo Wu^#^

^*^These authors contributed equally to this study: Weinan Yang, Zihao An and Honglu Cai.

^#^Corresponding authors: Jianbin Xu, An Liu and Haobo Wu.

This PDF file includes:

Experimental Section

Supplementary Figures. 1-25

Supplementary Tables. 1-9

Supplementary References. 1-23

**Experimental Section**

**Cellular and bacterial culture**

Bone marrow mesenchymal stem cells (BMSCs) were isolated from the bone marrow of 4-week-old C57BL/6 mice according to previously reported protocols^1^, which were cultured in α-MEM medium (12571063, Gibco) supplemented with 10% fetal bovine serum (FBS, A5256701, Gibco) and 1% penicillin/streptomycin (15140122, Gibco) with 5% CO_2_ at 37 ℃. Primary bone marrow-derived macrophages (BMDMs) were isolated from the femurs and tibiae of C57BL/6 mice. Briefly, 8-week-old C57BL/6 mice were aseptically dissected, femurs and tibiae were harvested, and the medullary cavity was flushed. BMDMs were cultured in medium containing 30 ng/mL macrophage colony-stimulating factor (M-CSF). After 3 days of culture, the cells were used for further experiments. To generate immunosuppressive macrophages, 20 ng/mL IL-4 (PeproTech, USA) was added to the medium. J774A.1 and RAW 264.7 macrophages were obtained from the Cell Collection of Chinese Academy of Sciences (Shanghai, China), which were cultured in high-glucose DMEM (11965167, Gibco) containing 10% FBS and 1% penicillin/streptomycin with 5% CO_2_ at 37 ℃. Gram-positive *S. aureus* strain (ATCC-25923), GFP- and RFP-labeled MRSA strains (USA300) were purchased from the American Type Culture Collection (ATCC), and cultured in LB broth medium (A507002-0250, Sangon Biotech) at 37 ℃.

**Animals**

Eight-week-old female C57BL/6 mice were procured from Vital River Laboratory Animal Technology Co. (Beijing, China). The present study was conducted in accordance with the ARRIVE guidelines for reporting animal experiments and received approval from the Animal Ethics Committee of The Second Affiliated Hospital, Zhejiang University School of Medicine (SAHZU-2024-240). The study complies with the ARRIVE 2.0 reporting guidelines for in vivo animal research. Mice were housed under standard conditions, including regulated room temperature (21 ± 2 ℃), a 12-hour dark/light cycle with free access to water and irradiated food. The housing environment maintained a pathogen-free setting with relative humidity between 45% and 65%. Anesthesia with 1-2% isoflurane in oxygen via a nose cone was used during experiments, and carbon dioxide asphyxiation was employed for euthanasia.

Lyz2-Cre mice, which express codon-optimized Cre recombinase under the control of the mouse Lyz2 endogenous promoter, were obtained from the Jackson Laboratory (strain number 004781). Gls-flox mice (Gls^fl/fl^, strain number NM-CKO-200181, C57BL/6 genetic background) were purchased from the Shanghai Model Organisms Center, Inc. (Shanghai, China). Macrophage-specific Gls-knockout mice (Gls^CKO^) were subsequently produced by intercrossing Gls^fl/fl^ mice with Lyz2-Cre mice. Eight-week-old mice were used in the studies, with littermates serving as controls.

**Mouse model of IBI caused by *S. aureus***

The mouse model of IBIs was established as per previously reported protocol with minor modifications^2, 3^. All handling and surgical procedures followed the approved guidelines. The C57BL/6 mice were deeply anesthetized and the right legs were manually disinfected and depilated. After layer-by-layer incisions of the knee joints, the tibia was exposed by blunt dissection and a unicortical hole was created by a 27-gauge syringe needle. Subsequently, 10 µL of 1 × 10^8^ CFU/mL MRSA were injected into the bone cavity using a micro-syringe, followed by the placement of a custom-made titanium rod implant. In the end, the incision was closed with sutures.

**Bulk RNA-seq analysis**

Bulk RNA sequencing data analysis was performed using three biological replicates (GSM5073659, GSM5073660, GSM5073661, GSM5073662, GSM5073663, GSM5073664, GSM5073665, GSM5073666, GSM5073667, GSM5073668, GSM5073669, GSM5073670) in each group from the GEO repository GSE166522^4^. All downstream transcriptomic data analyses were conducted using R language. Genes with a total expression value of zero across all samples were filtered out from the gene expression matrix before downstream analysis. Principal component analysis (PCA) was performed to evaluate the reliability of sample grouping and the consistency among biological replicates within each group. Differential gene expression analysis was conducted using the DESeq2 package. Log_2_ fold change was used to measure the magnitude of expression differences. The resulting *p* values were adjusted using the Benjamini–Hochberg correction method. Genes with an adjusted *p* value < 0.05 and an absolute log_2_ fold change ≥ 1.5 were defined as differentially expressed genes.

Differentially expressed genes were visualized using heat maps and volcano plots generated with the ComplexHeatmap and ggplot2 packages^5^. Gene Ontology (GO) enrichment analysis, including biological process, cellular component, and molecular function categories, and Kyoto Encyclopedia of Genes and Genomes pathway enrichment analysis were performed using the clusterProfiler package^6^. Gene Set Enrichment Analysis was also performed using the GSEA function in clusterProfiler. Gene set variation analysis was conducted using the GSVA package, with the kcdf parameter set to “Gaussian” and the method parameter set to "gsva"^7^. A *p* value < 0.05 and a false discovery rate (FDR) < 0.1 were considered statistically significant for enrichment analyses. Enrichment results were visualized using ggplot2 package.

**Single-cell RNA-seq data preprocessing and quality control**

Single-cell RNA sequencing data analysis was performed using three biological replicates (GSM7746354, GSM7746355, GSM7746356), including 3, 7 and 14 days post-infection (dpi) / 2 weeks post-infection (wpi), from the GEO repository GSE242039^8^. Raw 10X Genomics count matrices were imported using Read10X and processed with Seurat. During Seurat object creation, cells with fewer than 200 detected genes were excluded, and genes expressed in fewer than 5 cells were removed. The percentage of mitochondrial transcripts was calculated using genes matching the “^mt-” pattern. Ribosomal genes were identified using the “^Rp[sl]” pattern, and the percentage of ribosomal transcripts was also calculated for quality assessment. After merging datasets from different time points, cells with more than 2,500 detected genes or more than 5% mitochondrial transcripts were excluded from downstream analysis. The distributions of nCount_RNA, nFeature_RNA, percent.mt, and percent.rb before and after quality-control filtering were shown in Supplementary Fig. 1.

**Normalization, batch-effect correction, dimensionality reduction, and clustering**

The quality-filtered single-cell dataset was normalized using the LogNormalize method with a scale factor of 10,000. The top 2,000 highly variable genes were identified using the variance-stabilizing transformation method. The data were then scaled, and principal component analysis was performed based on the highly variable genes. To minimize potential batch effects across different injury time points, Harmony integration was performed using sample identity/time point (orig.ident) as the batch variable^9, 10^. The effectiveness of batch-effect correction was assessed by comparing PCA- and Harmony-based embeddings grouped by sample origin. The first 30 Harmony dimensions, selected based on the elbow plot, were used for downstream UMAP and t-SNE visualization, neighbor graph construction, and clustering. Clustering was performed using Seurat’s graph-based clustering algorithm at multiple resolutions. Resolution 1.0 was used for the main cell-type annotation. Cell identities were assigned based on canonical marker genes, including markers for macrophage/mononuclear phagocyte populations, neutrophils, T cells, B cells, NK cells, dendritic cells, basophils, and cycling cells. Cycling cells were identified based on canonical proliferation markers, including Mki67 and Top2a.

**Macrophage/mononuclear phagocyte subclustering and annotation**

Macrophage/mononuclear phagocyte populations were extracted from the annotated global single-cell dataset and reanalyzed using the SCP and Seurat workflow. The subset was normalized, scaled, and integrated using Harmony with orig.ident as the batch variable. Dimensionality reduction was then performed based on the Harmony-corrected embeddings. The first 30 Harmony dimensions were used for UMAP visualization, neighbor graph construction, and clustering. Macrophage subclustering was performed using Seurat’s graph-based clustering algorithm with a clustering resolution of 1.0. Macrophage subclusters were annotated according to cluster-enriched marker genes and canonical macrophage-associated markers (Fig. 1H). Specifically, the macrophage/mononuclear phagocyte compartment was divided into 11 subclusters. These annotations were supported by the expression of representative marker genes, including Icam1 for Icam1+_Mac, Nos2 for Nos2+_Mac, Cxcl2 for Cxcl2+_Mac, Il1b for Il1b_Mac, interferon-stimulated genes such as Ifit1, Ifit2, and Ifit3 for Ifit+_Mac, Tgfb1 for Tgfb1+_Mac, Camp for Camp+_Mac, Cd9 for Cd9+_Mac, Spp1 for SPP1+_Mac, Prx1 for Prx1+_Mac, and ribosomal protein genes for Rplhigh_Mac. The macrophage identity of these subclusters was further supported by broad expression of macrophage/mononuclear phagocyte-associated genes, including Ptprc, Cd68, Lyz1, Lyz2, and Adgre1. Cluster-enriched marker genes were identified using Seurat’s FindAllMarkers function with the Wilcoxon rank-sum test. For the initial global cell-type annotation, positive markers were identified using a log_2_ fold-change threshold of 0.25 and a minimum expression fraction of 0.2. For macrophage subcluster marker identification, a log-fold-change threshold of 0.5 and a minimum expression fraction of 0.2 were applied.

**Differential expression, enrichment, and macrophage polarization analyses**

Differential expression analysis among macrophage subclusters was further performed using RunDEtest from the SCP package. Adjusted P values were used to control for multiple testing. Genes with adjusted P values < 0.05 and average log2 fold change > log2(1.5) were selected for downstream Gene Ontology biological process enrichment analysis using the clusterProfiler package. Macrophage polarization-related signatures were evaluated through two complementary methodologies: Seurat's AddModuleScore assessed predefined proinflammatory (Nos2, Il6, Il12b, Tnf, Cxcl9, Cxcl10, Cd80, Cd86, Il1b) and immunosuppressive (Arg1, Mrc1, Cd163, Il10, Tgfb1) gene modules, while pathway activity was concurrently evaluated using AUCell algorithm (V1.32.0)^11^. Final visualizations were generated using Seurat (V5.4.0), SCP (V0.5.6), ggplot2 (V4.0.2), ComplexHeatmap (V2.26.1), RColorBrewer (V1.1), and related R packages^5^.

**Targeted metabolomics of** **human infected tissue samples**

A total of 20 infected tissue samples from IBI patients were collected from the Second Affiliated Hospital of Zhejiang University School of Medicine, who were diagnosed as *S. aureus* monomicrobial infection via bacterial culture identification or Next-Generation Sequencing (NGS). Besides, ten intra-articular soft tissue samples from total knee arthroplasty patients were used as controls. All handling and sampling procedures were approved by the Ethics Committee of the Second Affiliated Hospital, Zhejiang University School of Medicine (SAHZU-2024-1218).

After isolation from patients, infected tissue samples were thoroughly rinsed with normal saline until no blood was visible. The targeted metabolomics analysis of amino acid was performed on the Shengyang Biotech LC-MS/MS metabolomics platform (Jinan, China). In brief, a 200 mg sample of each patient was mixed with 800 μL ice-cold water. The mixture was vortexed for 5 min, extracted by sonication for 30 min, and then centrifuged at 4 ℃ for 10 min with a speed of 12,000 rpm to collect supernatants. Before LC-MS/MS, samples were randomized and blinded to avoid the impact of instrument fluctuations on the results. Chromatographic separation was performed on a Hypersil Gold column (100×2.1 mm, 1.9 μm) using a Vanquish UHPLC system (Thermo Fisher, Germany) with the following gradient: 0–1 min 3% B; 1–5 min 3–34% B; 5–6 min 34-90% B; 6–7 min 90% B; 7–8 min 90-3% B; 8-10 min 3% B. Mobile phase A was 0.1% formic acid in water. Mobile phase B was 0.1% formic acid in acetonitrile. The flow rate was set as 0.3 mL/min, and the injection volume was 1 µL. The column temperature and autosampler temperature were set to 35 ℃ and 4 ℃, respectively. A quality control (QC) sample was set at regular intervals among the experimental samples in the sample cohort to detect and evaluate the stability and repeatability of the system. A mixture of amino acid metabolite standards was set in the sample cohort to correct the chromatographic retention time. Mass data acquisition was performed using an AB SCIEX QTRAP 5500 mass spectrometer (AB SCIEX, USA) in multiple reaction monitoring (MRM) mode to monitor 22 endogenous water-soluble metabolites. Chromatogram review and peak area integration were performed using Analyst and SCIEX OS (AB SCIEX, USA). Metabolite identification was performed by correcting retention times using amino acid standards. The processed data were exported for further analysis.

**In vitro** **macrophage infection model**

The macrophage infection model was established as described previously^12-14^. Briefly, J774A.1 or RAW 264.7 cells were firstly seeded in 12-well plates with high-glucose DMEM (11965092, Gibco) to promote cell proliferation. Afterward, the culture medium was exchanged with the antibiotic-free and glutamine-free high-glucose DMEM (11960077, Gibco) and the cells were infected with GFP-labeled MRSA at a multiplicity of infection (MOI) of 20 for 2 h. Afterward, cells were washed with PBS and gentamicin (40 μg/mL) was added for 2 h to kill all residual extracellular bacteria. Next, cells were washed with PBS and incubated with glutamine-free high-glucose DMEM for further experiments.

**RNA sequencing**

Macrophage infection model was established using RAW 264.7 cells and the GFP-labeled MRSA strain (USA300). The cells infected with GFP-labeled MRSA were harvested at different infection time points by sorting out GFP-positive cells by fluorescence-activated cell sorting (FACS). Then, total RNA of the cells was extracted with the addition of Trizol reagent (Invitrogen). The samples were frozen in liquid nitrogen immediately. The samples were then subjected to sequencing using OE Biotech transcriptomics platform (Shanghai, China). The collected data were analyzed using the OE Cloud software.

**Flow cytometry analysis**

The detailed immune profiles were further analyzed using flow cytometry in vitro or in vivo. The cell suspension was preblocked with CD16/32 antibodies for 10 min at room temperature. The cells were then stained with flow cytometry antibodies (Supplementary Table 4) according to the manufacturer’s instructions. For intracellular staining, the cell suspension was fixed and permeabilized with Cyto-Fast™ Fix/Perm Buffer Set (426803, Biolegend) after the surface antibody staining. Flow cytometry analysis was performed using Beckman Coulter CytoFLEX LX and CytExpert v2.4 software, and flow data were analyzed via FlowJo v10.

**Fluorescence-activated cell sorting**

The bone marrow was extruded and filtered through a 70 μm cell strainer to harvest single-cell suspension. Red blood cells (RBCs) were lysed by Red Blood Cell Lysis Buffer (R1010, Solarbio). The CD45 positive immune cells or peri-implant macrophages (CD45^+^ CD11b^+^ F4/80^+^ cells) were sorted out from peri-implant bone marrow through FACS method after different treatments.

**Neutrophil depletion**

For in vivo neutrophil depletion, mice received an intraperitoneal injection of anti-mouse Ly6G antibody clone 1A8 (Bio X Cell, Cat# BE0075-1) or rat IgG2a isotype control clone 2A3 (Bio X Cell, Cat# BE0089) at 10 μg/g body weight per mouse one day before infection. On day 0, the IBI model was established, followed by an additional intraperitoneal injection. A third intraperitoneal injection of antibody was administered on day 2. The neutrophil depletion efficiency was confirmed by flow cytometry.

**Glutamate and glutamine assay**

Intracellular levels of glutamate and glutamine in CD45 positive immune cells or macrophages (10^5^ cells per sample) were analyzed by bioluminescent assay via Glutamine/Glutamate-Glo™ Assay (J8021, Promega) according the manufacturer’s procedure.

**ELISA analysis**

The levels of IL-6, IL-10, TNF-α (RK00008, RK04490 and RK00027, respectively, ABclonal) and IFN-γ (EK0375, Sciencell) in peri-implant bone marrow aspirates or cell samples were detected using ELISA kits following the manufacturer’s procedure.

**Stable isotope-assisted tracing and fluxomics**

To investigate mitochondrial anaplerosis from glutamine in infected macrophages, the macrophage infection model was established in aforementioned manners. At 0hpi and 6.5dpi, cells were washed with PBS, and the medium was changed with glutamine-free and glucose-free DMEM (A1443001, Thermo Fisher Scientific) containing 10% FBS, 25 mM glucose (63005518, Sinopharm Chemical Reagent Co., Ltd) and 8 mM [U-^13^C] glutamine (CLM-1822-H-0.25, Cambridge Isotope Laboratories, Inc.). The cells were collected for stable isotope-labeled metabolic tracing after 12 h of incubation.

The TCA cycle metabolite levels was subjected to commercial metabolic flux analysis (Metabo-Profile Biotech Co., Ltd, Shanghai, China). The cell samples were mixed with 500 μL of cold 80% extraction solvent and sonicated for 10 min and centrifuged at 18,000 g for 15 min at 4 °C. For LC–MS, samples were dried under nitrogen flow and reconstituted in methanol-water (1:1, v/v) for injection using a UPLC Acquity (Waters, USA) separation system coupled with a Xevo TQS (Waters, USA). Samples were injected onto a UPLC BEH C18 column (100 mm × 2.1 mm, 1.7 µm) using a linear gradient at a flow rate of 0.3 mL/min. The eluents for the mobile phases were eluent A (0.1% formic acid in water) and eluent B (0.1% formic acid-methanol). The solvent gradient was set as follows: 5% B, 1 min; 5%-10% B, 0.5 min; 10%-100% B, 7 min; 100% B, 2 min; 100%-5% B, 0.5 min; 5% B, 1 min. Raw data generated by UPLC-TQS were processed using Waters MassLynx software (v4.1, Waters, USA) for peak extraction, integration, identification, and quantitative analysis of each metabolite. Correction for natural abundance was required prior to meaningful interpretation. The correction used both a generally applicable correction matrix and an isotopic correction matrix generated from unlabeled control samples. Metabolite mass isotopomer distribution (MID, they are also called mass distribution vector, MDV) was determined based on the ratio of the integrated peak areas of each isotopomer to the sum of all the integrated peak areas of the possible isotopomers for each given metabolite.

**Quantitative real-time polymerase chain reaction**

Cells were lyzed on ice using 300 µL of TRIzol reagent (15596018, Invitrogen) for 15 min. 60 µL of chloroform (10006818, Sinopharm Chemical Reagent Co., Ltd, China) was added to the TRIzol reagent and vortex for 10 s followed by centrifugation at 12,000 g for 15 min and collecting the supernatant containing RNA. Then, 150 µL isopropanol (80109218, Sinopharm Chemical Reagent Co., Ltd, China) was added to the supernatant and the mixture was kept at -20 °C overnight after vortex for 10 s. The next day, the sample was centrifuged at 12,000 g for 15 min to get the RNA, and the RNA was washed using 70% ethanol (10009218, Sinopharm Chemical Reagent Co., Ltd, China) two times and centrifuged at 7500 g for 10 min to obtain purified RNA. 20 µL RNAse-free water was used to dissolve RNA and the concentration was measured using Nanodrop 2000 spectrophotometer (Thermo Fisher Scientific). Next, 1 µg of mRNA was reverse-transcribed using 5X Evo M-MLV RT Master Mix for qPCR (AG11706, Accurate Biotechnology, China) using a 20 µL system to get cDNA. Finally, 180 µL RNAse-free water was used to dilute the cDNA.

For quantitative real-time polymerase chain reaction, a 20 µL system was used: 2 µL cDNA, 3 µL primer, 5 µL RNAse-free water and 10 µL 2X SYBR Green Pro Taq HS Premix (Rox Plus, AG11718, Accurate Biotechnology, China). The thermal cycling conditions were 95 °C for 1 min followed by 40 cycles of 95 °C for 5 seconds and 60 °C for 30 seconds using an Applied Biosystem^TM^ 7500 system (Thermo Fisher Scientific). Following the reactions, the raw data were analyzed using Real-Time PCR Software (Applied Biosystem, version 2.4). The data were then normalized to housekeeping gene (β-actin for cells) expression. Relative expression values were calculated using the 2^-ΔΔCt^ method and used for statistical analysis. All primer sequences are listed in Supplementary Table 5.

**Investigating the role of glutaminolysis-fueled anaplerosis in macrophage antibacterial** **activity**

Prior to infection with *S. aureus*, the macrophages were pretreated with glutamine or small molecules, and this treatment was continued until 7dpi (Supplementary Table 7). For the evaluation of intracellular ROS and mtROS, the treated cells were stained with CellROX™ Deep Red (C10422, Thermo Fisher Scientific, USA) and MitoTracker Red CM-H_2_Xros (M7513, Thermo Fisher Scientific, USA) for 30 min in the dark respectively. After co-incubation for indicated time, cells were stained with Hoechst (Beyotime, China), rinsed three times with PBS, observed using confocal laser scanning microscopy (CLSM, ZEISS LSM 710, Germany) with the presence of ProLong Live Antifade Reagent (P36975, Thermo Fisher Scientific, USA) and quantified using flow cytometry analysis. The phenotypes of macrophages were evaluated by flow cytometry in aforementioned manners. The flow cytometry antibodies used in this study are showed in Supplementary Table 4.

**Western blot analysis**

Total protein from treated cells was extracted using RIPA lysis buffer, and the concentration was determined by a BCA protein quantification kit. Proteins with different molecular weights were separated by SDS-PAGE, and then the protein bands were transferred to PVDF membranes. The membranes were blocked at room temperature for 1 h, washed three times with TBST for 5 min each time, incubated overnight at 4 ℃ with primary antibody against the target protein, washed 3 times with TBST, incubated at 4 ℃ for 2 h with horseradish peroxidase-labeled secondary antibody, washed 3 times with TBST, and developed by an ECL chemiluminescence reagent kit. The western blot antibodies used in this study are showed in Supplementary Table 7.

**Extraction and purification of OMVs**

*E. coli* BL21 (ΔmsbB) was constructed via CRISPR-mediated gene knockout through Inovogen Tech. Co. (Chongqing, China). Bacterial cultures were initiated by inoculating LB medium with 1% (v/v) of the strain and incubated with shaking until reaching logarithmic phase (OD_600_ = 1.0). Cells were pelleted by centrifugation at 4, 000 g for 15 min, and supernatants were filtered through 0.45 μm filters (Millipore brand) to remove residual bacteria. OMVs were sequentially enriched by ultracentrifugation (Beckman) at 150,000×g for 2 h at 4 °C. Purified OMVs were resuspended in PBS for downstream applications. Polymyxin B (PMB, an LPS-neutralizing antibiotic) was added to the OMV suspension to a final concentration of 100 μg/mL and incubated at room temperature for 2 h with gentle mixing. The PMB-treated OMV suspension was ultracentrifuged again at 150,000×g for 2 h at 4 °C to remove unbound PMB and impurities. The pelleted material was resuspended in PBS to prepare the OMV stock solution (samples designated for zeta potential analysis were resuspended in double-distilled water). The OMV concentration, defined by the protein concentration of the preparation, was finally quantified using a bicinchoninic acid (BCA) protein assay kit.

**Synthesis and characterization of DSPE-PEG_2000_-Hyd-MTP**

The DSPE-PEG_2000_-Hyd-MTP was synthesized by Xi’an Ruixi Biological Technology (China). Specifically, the mannose-functionalized antibacterial peptides (MTP, CRLYLRIGRR) were synthesized via solid-phase synthesis. Then, 100 mg DSPE-PEG_2000_-Hyd-NHS, 1.1 equivalents MTP and 3.0 equivalents triethylamine were dissolved in dimethylformamide (DMF) and allowed react at room temperature for 12 h. Free unconjugated components were subsequently removed using Slide-A-Lyzer Dialysis Cassettes (2000 MWCO, Thermo Fisher Scientific, USA) to obtain DSPE-PEG_2000_-Hyd-MTP, the successful synthesis of which was confirmed by ^1^H NMR.

To assess the pH-responsive properties of DSPE-PEG_2000_-Hyd-MTP, the DSPE-PEG_2000_-Hyd-MTP was incubated in PBS buffer at three different pH levels (pH 7.4, pH 6.5 and pH 5.5) for 12 h. After freeze-drying, Fourier transform infrared spectrophotometer (FTIR) spectra of these samples were recorded on an FTIR spectrometer (Nicolet 6700, Thermo Fisher Scientific, USA) to analyze chemical groups of samples.

**Preparation of mGls@HEV-MTP**

The EGFP-encoding chemically modified mRNA (R1011, APExBIO, Houston, USA) was used as a reporter mRNA for corresponding experiments, and the noncoding EGFP mRNA without start codon (R1031, APExBIO, Houston, USA) was used as a negative control. The *Gls* gene sequence was obtained from GenBank, and the *Gls*-encoding chemically modified mRNA was synthesized by APExBIO (Houston, USA). The *Gls* mRNA was polyadenylated, optimized for mammalian systems, and modified with Cap1 capping, ribonucleobases pseudouridine (ψU) and 5-methylcytidine (5-meC) to reduce nonspecific immune stimulation^15^. A noncoding scrambled mRNA control was produced with an equivalent length and nucleotide composition with the same chemical modifications as our *Gls* mRNA. Following the strategy reported by Brook et al., the canonical start codon was replaced with a stop codon, and the final scrambled sequence contained no start codon to minimize unintended translation^16^. The control HEV-MTP was loaded with the noncoding mRNA control at an equivalent mRNA dose to that of coding mRNA.

To synthesize mRNA-loaded lipid nanoparticles (mRNA@LNPs), a microfluidic device was used to mix the aqueous phase containing mRNA with the ethanol phase containing lipids and cholesterol. Briefly, an ethanol phase was prepared containing hydrogenated soybean phospholipids (HSPC), 1,2-distearoyl-sn-glycero-3-phosphoethanolamine-N-[carboxy(polyethyleneglycol)-2000] (DSPE-PEG_2000_), high-purity cholesterol (CHO-HP), 1,2-dioleoyl-3-trimethylammonium-propane chloride (DOTAP), ionizable lipid (SM-102) at a precise molar ratio of 29.4:2.6:20:20:28. An aqueous phase containing mRNA in 50 mM citrate buffer (pH 4.0) was prepared separately. The ethanol and aqueous phases were subsequently combined in a microfluidic device with a staggered herringbone mixer architecture at a volume ratio of 1:3 to generate the mRNA@LNPs. To remove any remaining organic solvent and free compounds, the mRNA@LNPs were washed with ice-cold DNA/RNase-free water using an Amicon Ultra centrifugal filter (molecular weight cut-off, 100 kDa; Millipore). Finally, the mRNA@LNPs were concentrated in 1 mL of fresh PBS, and were stored at 4 °C until further use.

For the preparation of mRNA-loaded hybrid vesicles (mRNA@HEVs), mRNA@LNPs were hybridized with OMVs at various mass ratios via filter membranes with pore sizes of 400, 200 and 100 nm using a mini-extruder, and uncoated membranes were removed through centrifugation and washed until no protein was detected in the supernatant. Finally, DSPE-PEG_2000_-Hyd-MTP was inserted into the mRNA@HEVs, and mRNA@HEVs-MTP was obtained after removing free DSPE-PEG_2000_-Hyd-MTP.

**Supplementary *Gls* mRNA coding sequence**

ATGATGAGACTGAGAGGCAGCGCCATGCTGAGAGAGCTGCTGCTGAGACCTCCTGCCGCCGTGGGCGCTGTGCTGAGAAGAGCCCAGCCTCTGGGCACCCTGTGCAGGAGACCTAGAGGCGGCTCCAGACCTACCGCCGGCCTGGTTGCCGCCGCTAGACTGCACCCTTGGTGGGGCGGCGGCGGAAGAGCTAAGGGACCTGGCGCCGGCGGCCTGTCTTCCTCTCCTTCCGAGATCCTGCAGGAGCTGGGCAAGGGCGGCACCCCTCCACAGCAGCAGCAGCAACAGCAGCAGCAGCCTGGCGCCTCCCCACCAGCTGCTCCAGGACCTAAGGATAGCCCCGGCGAGACCGATGCCTTCGGCAACAGCGAGGGCAAGGAGATGGTGGCCGCCGGCGACAACAAGATCAAGCAGGGCCTGCTGCCCAGCCTGGAGGACCTGCTGTTCTACACAATCGCCGAGGGCCAGGAGAAGATCCCCGTGCACAAGTTCATCACCGCCCTGAAGAGCACAGGCCTGAGGACCTCCGACCCCAGACTGAAGGAGTGCATGGACATGCTGAGACTGACACTGCAGACCACCTCCGACGGCGTGATGCTGGATAAGGATCTGTTCAAGAAGTGCGTGCAGAGCAACATCGTGCTGCTGACACAGGCCTTCAGAAGGAAGTTCGTGATCCCCGACTTCATGAGCTTCACCAGCCACATCGATGAGCTGTACGAGTCCGCCAAGAAGCAGAGCGGCGGCAAGGTGGCCGACTACATCCCTCAGCTGGCCAAGTTCAGCCCTGATCTGTGGGGCGTGAGCGTGTGTACAGTGGATGGCCAGAGGCACTCCATCGGCGATACCAAGGTGCCTTTCTGCCTGCAGAGCTGCGTGAAGCCCCTGAAGTACGCCATCGCCGTGAACGACCTGGGCACCGAGTACGTGCACAGATACGTGGGCAAGGAGCCTTCCGGCCTGAGGTTCAACAAGCTGTTCCTGAACGAGGATGATAAGCCCCACAACCCTATGGTGAACGCCGGCGCCATCGTGGTGACAAGCCTGATCAAGCAGGGAGTGAACAACGCCGAGAAGTTCGATTACGTGATGCAGTTCCTGAACAAGATGGCCGGCAACGAGTACGTGGGCTTCAGCAACGCCACCTTCCAGAGCGAGAGAGAGTCCGGCGATAGAAACTTCGCCATCGGCTACTACCTGAAGGAGAAGAAGTGTTTCCCCGAGGGCACCGACATGGTGGGCATCCTGGACTTCTACTTCCAGCTGTGTAGCATCGAGGTGACATGCGAGAGCGCCTCCGTGATGGCCGCCACCCTGGCTAACGGCGGCTTCTGCCCTATCACAGGCGAGAGGGTGCTGTCCCCAGAGGCCGTGAGAAACACCCTGTCCCTGATGCACAGCTGCGGCATGTACGACTTCTCCGGCCAGTTCGCCTTCCACGTGGGCCTGCCCGCCAAGAGCGGCGTTGCTGGAGGCATCCTGCTGGTGGTGCCCAACGTGATGGGCATGATGTGTTGGTCCCCTCCCCTGGACAAGATGGGCAACAGCGTGAAGGGCATCCACTTCTGCCACGATCTGGTGAGCCTGTGCAACTTCCACAACTACGACAACCTGAGACACTTCGCCAAGAAGCTGGACCCCAGGAGGGAGGGCGGCGATCAGAGAGTGAAGAGCGTGATCAACCTGCTGTTCGCCGCCTACACAGGCGATGTGAGCGCCCTGAGAAGATTCGCCCTGTCCGCTATGGACATGGAGCAGAGAGATTACGACAGCAGGACAGCCCTGCACGTGGCCGCCGCTGAGGGACATGTGGAGGTGGTGAAGTTCCTGCTGGAGGCCTGTAAGGTGAACCCTTTCCCCAAGGACAGATGGAACAACACACCTATGGACGAGGCCCTGCACTTCGGCCACCACGACGTGTTCAAGATCCTGCAGGAATACCAGGTGCAGTACACCCCTCAGGGCGACAGCGACGACGGCAAGGGCAACCAGACCGTGCACAAGAACCTGGATGGCCTGCTGTGA

**Characterization of mGls@HEV-MTP**

The transmission electron microscope (TEM, JEM-1400) was used to characterize the morphology of nanovesicles. Dynamic light scattering (DLS) with a Zetasizer Nano ZS instrument (Nano ZS90 Zetasizer, Malvern) was used to characterize the size distribution and zeta-potential values of nanovesicles. The UV-vis absorbance of different nanovesicles was measured by SHIMADZU UV-3600 Plus spectrophotometer (Japan). The final mRNA concentration and encapsulation efficiency were measured by Quant-it Ribogreen Assay Kit (Thermo Fisher Scientific), and the encapsulation efficiency was approximately 93.1%.

Furthermore, to evaluate the mRNA protection abilities of different nanovesicles, free *Gls* mRNA, mGls@LNP, and mGls@HEV-MTP were incubated in PBS (pH 7.4) with 50% FBS at 37 °C. At various predetermined time points (0, 6, 12, 18, 24, 36 and 48 h), the samples were lysed with Triton X-100 to release the mRNA, followed by analysis through electrophoresis on 2% agarose gels.

The in vitro cytotoxicity of nanoparticles was assessed using a CCK-8 assay. Briefly, J774A.1 cells were seeded into 96-well plates at a density of 10^4^ cells per well and incubated overnight. Subsequently, the cells were treated with mGls@HEV-MTP at the indicated concentrations (ranging from 2 to 12 μg/mL) for 24 h, after which the CCK-8 assays were conducted according to the manufacturer’s instructions.

**In vitro cellular uptake of nanovesicles**

To assess cellular uptake, J774A.1 cells and BMSCs were seeded on glass-bottom dishes (Biosharp). The nanovesicles, including mGls@LNP, mGls@HEV, HEV-MTP and mGls@HEV-MTP, were labeled with DID (red, Abmole, USA). Additionally, a DID-PBS control group was prepared by treating PBS with DID under the same condition used for the nanovesicle labeling to exclude any potential effects from residual dye. The DID-labeled nanovesicles (5 μg/mL) were co-incubated with J774A.1 cells for 0.5 h and BMSCs for 1 h at 37 °C, respectively, followed by thorough washing to remove extracellular nanovesicles. After incubation, the cells were fixed with 4% paraformaldehyde for 10 min. Nuclei were stained with DAPI (blue, Boster, China), and cell membranes were stained with phalloidin (green, Beyotime, China). Nanovesicle internalization was visualized using confocal laser scanning microscopy (CLSM, ZEISS LSM 710, Germany). To achieve absolutely quantify and analyze the kinetics of nanovesicle uptake, J774A.1 cells were harvested at multiple time points (6, 12 and 24 h), washed thoroughly to remove any extracellular nanovesicles, and analyzed using flow cytometry (Beckman Coulter, USA). Data were processed with FlowJo software (version 10).

**In vivo biodistribution of nanovesicles**

For the biodistribution study, IBI model mice were randomly divided into two groups and administered DID-labeled mGls@HEV or mGls@HEV-MTP (5 mg protein/kg) via intravenous injection. At 1dpi and 2dpi, anesthetized mice (maintained with 5% isoflurane/oxygen) were imaged using an in vivo imaging system (PerkinElmer) with 675 nm excitation and 740 nm emission wavelengths.

**In vivo analysis of** **nanovesicle uptake by different cells**

The nanovesicle uptake by different cell populations in vivo was analyzed using flow cytometry. Briefly, IBI model mice were administered DID-labeled mGls@LNP or DID-labeled mGls@HEV-MTP via tail vein injection at a dose of 5 mg protein/kg. At 24 h post-injection, peri-implant bone marrow was collected for flow cytometry analysis.

**pH-responsive hierarchically selective delivery of** **mGls@HEV-MTP**

To assess macrophage uptake of mGls@HEV-MTP at different pH values, J774A.1 cells were seeded in six-well plates at a density of 10^5^/well each and incubated at 37 °C until 50-60% confluence was observed. GFP-labeled MRSA and DID-labeled mGls@HEV-MTP were co-incubated in DMEM with 10% FBS (pH 7.4) and first observed under a fluorescence microscope after 5 min. Then, the pH was adjusted to 5.5 with hydrochloric acid solution and the samples were observed under a fluorescence microscope. Flow cytometry was used to quantify the uptake of nanovesicles by macrophages.

To assess MTP-induced bacterial phagocytosis by macrophages, GFP-labeled MRSA was firstly pretreated with mGls@HEV-MTP at pH 5.5 for 24 h. MRSA was collected by centrifugation at 5,000 rpm. Then, J774A.1 cells were co-incubated with pretreated GFP-labeled MRSA at a multiplicity of infection (MOI) of 20 for 1 h at 37 °C. After three washing steps using PBS, flow cytometry was used to quantify MTP-induced bacterial phagocytosis by macrophages.

**Nanovesicle-mediated mRNA translation efficiency in macrophages**

For visual evaluation of mRNA delivery, the *Gls* coding sequence was replaced with EGFP to generate EGFP mRNA-loaded nanovesicles. J774A.1 cells were incubated with various nanovesicles, including mEGFP@LNP (5 μg EGFP mRNA/mL), mEGFP@HEV (5 μg EGFP mRNA/mL), mEGFP@HEV-MTP (5 μg EGFP mRNA/mL) and HEV-MTP, for 24 h. EGFP expression was visualized by confocal microscopy. To quantify mRNA expression, J774A.1 cells were treated with various nanovesicles and analyzed by flow cytometry. Moreover, to evaluate the GLS1 expression efficiency and temporal dynamics in macrophages, J774A.1 cells were incubated with LNP, OMV, HEV-MTP and mGls@HEV-MTP (5 μg *Gls* mRNA/mL) at different time points (1 d, 2 d, 3 d and 5 d), and corresponding cell lysates were harvested for Western blot analysis.

**Evaluation of ROS and mtROS levels in macrophages**

The infected macrophages were performed with the indicated treatments for 7 days in glutamine-free DMEM with 10% FBS. For the evaluation of intracellular ROS and mtROS, the treated macrophages were stained with CellROX™ Deep Red (C10422, Thermo Fisher Scientific, USA) and MitoTracker Red CM-H2Xros (M7513, Thermo Fisher Scientific, USA) respectively, for 30 min in the dark. After co-incubation for the indicated time, cells were stained with Hoechst (Beyotime, China), rinsed three times with PBS, observed using confocal laser scanning microscopy (CLSM, ZEISS LSM 710, Germany) with the addition of ProLong Live Antifade Reagent (P36975, Thermo Fisher Scientific, USA) and quantified by flow cytometry analysis.

**In vivo** **therapeutic efficacy evaluation**

At 1dpi, IBI model mice were randomly divided into four groups with comparable body weights and received the intravenous injection of PBS (5 mL/kg), HEV-MTP (5 mg protein/kg), mGls@HEV (5 mg protein/kg) and mGls@HEV-MTP (5 mg protein/kg) every other day. Body weights, knee perimeters and joint angles were recorded at the indicated time points. At 14dpi, IBI model mice were euthanized and the tibiae were collected for histological analysis. Based on the gross bone pathological score (GBPS, Supplementary Table 8), the macroscopic evaluation of the infected tibial specimens was conducted independently by 3 experienced observers who were blinded to the group allocation, and the average value of the three scores was adopted for analysis. Hematoxylin and Eosin (H&E) staining kit (K1142, APExBIO, Houston, USA) and Gram staining solution (G1065, Servicebio, China) were used to evaluate inflammation and bacterial load. According to the histological scores (Supplementary Table 9) from the H&E staining images, the severity of the infection was evaluated independently by 3 experienced observers who were blinded to the group, and the average value of the three scores was used for analysis. Blood samples were collected for routine blood test using a TEK8500 VET Automatic Hematology Analyzer (TECOM, China) via retro-orbital blood collection under anesthesia.

At 14dpi, the bone marrow suspensions and titanium rod implants were harvested, and bacterial burden was assessed by colony-forming unit (CFU) counting. Specifically, the tibia bone marrow suspension was resuspended in 1 mL of PBS for intracellular and extracellular bacterial colony counting after RBC lysis.

The implants were placed in a six-well plate and fixed with 2.5% glutaraldehyde at 4 °C overnight. Next, the implants were placed in graded ethanol solutions for dehydration (50%, 60%, 70%, 80%, 90% and anhydrous ethanol) for 10 min, freeze-dried, and coated with platinum. Then the biofilms on the implant surfaces were observed using a field-emission SEM (IGMA 300, Zeiss, Germany).

**Study design**

Based on previous studies, the bacterial burden in the infection sites, expressed as log_10_CFU/mL, was prespecified as the primary outcome for the in vivo experiments^3, 17, 18^. Based on our preliminary data and comparable prior experiments, we assumed a minimum biologically relevant difference of 1.3 log_10_CFU/mL and a standard deviation of 0.7 log_10_CFU/mL. With a two-sided α of 0.05 and 80% power, the calculated sample size was rounded up to five animals per group. Investigators responsible for treatment administration and statistical analysis were blinded to the group allocation. The group allocation codes were disclosed only after completion of the analyses.

**Statistical analysis**

All quantitative biological experiments were performed using independent biological replicates unless otherwise stated. For in vitro cellular experiments, one independent biological replicate referred to an independently cultured cell sample subjected to the indicated treatment from a separate experiment. For in vivo experiments, one independent biological replicate referred to one animal. For human-derived sample analyses, each independent included patient represented one independent biological replicate. All quantitative experiments for nanomaterial characterization were performed using independent material sample replicates unless otherwise stated. The exact *n* values were provided in the figure legends. All data were presented as mean ± standard deviation. The normality of the data was assessed using the Shapiro-Wilk test. Unless otherwise specified, all statistical tests were two-sided. For comparisons between two groups, the Student’s t test was used for normally distributed data, and the Mann-Whitney U test was used for normally distributed data. For comparisons among multiple groups, one-way analysis of variance (ANOVA) followed by Tukey’s post hoc test was used for normally distributed data, and the Kruskal–Wallis test followed by Dunn’s multiple-comparison test was used for non-normally distributed data. All statistical tests used are specified in the figure legends. All calculations and statistical analyses were conducted with GraphPad Prism 9. A *p*-value of < 0.05 was deemed statistically significant.

**Supplementary Figures**

**Supplementary Figure 1.** (**A**) Quality control metrics before and (**B**) after filtering of single-cell RNA-seq data. Violin plots showing the distributions of nCount_RNA, nFeature_RNA, percent.mt, and percent.rb across samples at 3dpi, 7dpi, and 2wpi.

**Supplementary Figure 2. Subclustering analysis of macrophage heterogeneity in *S. aureus* IBIs.** (**A**) UMAP visualization of CD45^+^ immune cell compartment in peri-implant tissue, stratified by three infection stages (3, 7, and 14dpi). (**B**) Volcano plots identifying differentially expressed genes (|log_2_ FC| > 0.5, adjusted p < 0.05) across macrophage subclusters.

**Supplementary Figure 3.** (**A**) GO enrichment analysis of DEGs among different macrophage subsets. (**B**) Dual-method quantification of proinflammation or immunosuppression polarization states: module scores computed via Seurat's AddModuleScore algorithm and pathway activity scores derived from AUCell analysis.

**Supplementary Figure 4.** Temporal expression profiles of glutamine metabolism-associated genes in proinflammatory macrophage subtypes (3, 7, 14dpi).

**Supplementary Figure 5.** (**A**) PCA of DEGs determined by RNA transcriptomic sequencing of infected RAW 264.7 at 3dpi and 2wpi. (**B**) Heatmap and (**C**) volcano plot of DEGs in infected RAW 264.7 cells at 3dpi and 2wpi. (**D**) Combination of the unsupervised hierarchical cluster and the inflection point method were employed to determine the optimal number of clustering (k = 8). (**E**) Temporal trend analysis of 15,153 genes in infected RAW 264.7 cells clustered into 8 patterns by Mfuzz-based fuzzy clustering and enrichment analysis.

**Supplementary Figure 6. The public dataset analysis of GSE166522.** (**A**) PCA of DEGs determined by RNA transcriptomic sequencing of IBI model mice at 3dpi and 2wpi. (**B**) Heatmap of DEGs of IBI model mice at 3dpi and 2wpi. (**C**) Volcano plot showed DEGs between IBI model mice at 3dpi and 2wpi. (**D**) GSEA analyses for the enriched pathways associated with positive regulation of the immune process in GSE166522. (**E**) Heatmap of mRNA transcriptional profiling depicted the DEGs involved in immune responses (*n =* 3). (**F**) GSEA analyses for the enriched pathways associated with glutamine metabolism in GSE166522.

**Supplementary Figure 7. Gating strategies for flow cytometry analysis.** Representative gating strategy of (**A**) peri-implant macrophages and (**B**) T cells in IBI model mice.

**Supplementary Figure 8.** Representative flow cytometry data of CellROX Deep Red staining to depict intracellular ROS levels of infected J774A.1 cells at different infection time points (*n =* 5). All *n* values indicated the number of independent biological replicates. All data were presented as the mean ± standard deviation.

**Supplementary Figure 9.** (**A**) Representative flow cytometry plots and quantification of intracellular residual *S. aureus*, (**B**) MitoTracker Red CM-H2Xros staining showing intracellular mtROS levels and (**C**) the immunophenotype of infected macrophages upon different treatments at 7dpi (*n* = 5). All *n* values indicated the number of independent biological replicates. All data were presented as the mean ± standard deviation. The *p*-values were determined by (**A-C**) one-way ANOVA with Tukey’s post hoc test for comparisons among multiple groups.

**Supplementary Figure 10.** Gene identification for Gls^CKO^ mice. (**A**) *Gls* homozygote is one band at 395bp. (**B**) Lyz2-Cre heterozygote is two bands at 350bp and 700bp.

**Supplementary Figure 11.** (**A**) Representative flow cytometry plots and quantification of intracellular residual *S. aureus* in differentially treated J774A.1 cells (*n =* 5). (**B**) Representative flow cytometry data of CellROX Deep Red staining to depict intracellular ROS levels of infected J774A.1 cells in the presence or absence of glutamine at 4hpi and 7dpi (*n =* 5). (**C**) Representative confocal fluorescence images of intracellular residual *S. aureus* and mtROS in infected J774A.1 cells. Hoechst was used to label the cell nucleus. Green fluorescence represented intracellular GFP-labeled MRSA. MitoTracker Red CM-H2Xros was used to label intracellular mtROS. Scale bar, 20 μm. (**D**) Representative flow cytometry data of MitoTracker Red CM-H_2_Xros staining showing intracellular mtROS levels of infected J774A.1 cells in the presence or absence of glutamine at different infection time points (*n =* 5). (**E**) Representative flow cytometry plots and quantification of phenotypes of infected J774A.1 cells in the presence or absence of glutamine (*n =* 5). (**F**) Western blotting of GLS1and β-actin in infected J774A.1 cells in the presence or absence of glutamine. (**G**) Relative expression of *Gls* in infected J774A.1 cells in the presence or absence of glutamine (*n =* 5). All *n* values indicated the number of independent biological replicates. All data were presented as the mean ± standard deviation. The *p*-values were determined by the Student’s t test for comparisons between two groups.

**Supplementary Figure 12.** Representative flow cytometry plots and quantification of macrophage phenotypes of infected RAW 264.7 cells in the presence or absence of glutamine (*n =* 5). All *n* values indicated the number of independent biological replicates. All data were presented as the mean ± standard deviation. The *p*-values were determined by the Student’s t test for comparisons between two groups.

**Supplementary Figure 13. Gating strategies for flow cytometry analysis.** Representative gating strategy of peri-implant macrophages and neutrophils in IBI model mice.

**Supplementary Figure 14.** (**A**) Flow cytometry analysis of intracellular mtROS, (**B**) total ROS and (**C**) surface CD63 levels of peri-implant neutrophils from Gls^fl/fl^ and Gls^CKO^ mice at 3dpi (*n* = 5). (**D**) Flow cytometry analysis of intracellular mtROS, (**E**) total ROS and (**F**) CD86 levels of peri-implant macrophages from Gls^fl/fl^ and Gls^CKO^ mice at 3dpi (*n* = 5). (**G**) Representative flow cytometry plots and quantitative analysis confirming neutrophil depletion in infection sites of mice after administration of a Ly6G antibody (*n* = 5). (**H**) Cultivation plates and (**I**) corresponding bacterial load quantification of the bone marrow aspirate from Gls^fl/fl^ and Gls^CKO^ mice receiving anti-mouse Ly6G antibody or isotype control antibody at 3dpi (*n* = 10). All *n* values indicated the number of independent biological replicates. All data were presented as the mean ± standard deviation. The *p*-values were determined by (**A-G)** the Student’s t test for comparisons between two groups, and (**I**) one-way ANOVA with Tukey’s post hoc test for comparisons among multiple groups.

**Supplementary Figure 15.** (**A**) Synthetic route of DSPE-PEG_2000_-Hyd-MTP. (**B**) ^1^H NMR of DSPE-PEG_2000_-Hyd-MTP and MTP.

**Supplementary Figure 16.** (**A**) SDS-PAGE analysis of membrane protein profiles of mGls@LNP, OMV, mGls@HEV and mGls@HEV-MTP. (**B**) Particle size variation trend of mGls@HEV-MTP in 10% FBS DMEM solution at 4 °C over one week (*n =* 3). All *n* values indicated the number of independent material sample replicates. All data were presented as the mean ± standard deviation.

**Supplementary Figure 17.** (**A**) Representative flow cytometry plots and quantification of iNOS and (**B**) CD86 levels of macrophages after treatment with various concentrations of HEV-MTP (*n* = 5). All *n* values indicated the number of independent biological replicates. All data were presented as the mean ± standard deviation. The *p*-values were determined by (**A, B**) one-way ANOVA with Tukey’s post hoc test for comparisons among multiple groups.

**Supplementary Figure 18.** Representative flow cytometry plots and quantification depicting the internalization of different nanovesicles by macrophages at various time points (*n =* 3). All *n* values indicated the number of independent biological replicates. All data were presented as the mean ± standard deviation.

**Supplementary Figure 19.** (**A**) Representative confocal images and (**B**) the fluorescence intensity of localization of GFP-labeled *S. aureus* (green) and DID-labeled nanovesicles (red). Scale bar, 100 μm.

**Supplementary Figure 20. Gating strategies for flow cytometry analysis.** Representative flow cytometry images of the gating strategy showing the percentage of various cells among DID^+^ cells in the infection sites in Fig. 5E.

**Supplementary Figure 21.** (**A**) Representative flow cytometry images of the gating strategy showing (**B**) the DID mean fluorescence intensity of various cells in the infection sites (*n* = 5). All *n* values indicated the number of independent biological replicates. All data were presented as the mean ± standard deviation. The *p*-values were determined by (**B**) one-way ANOVA with Tukey’s post hoc test for comparisons among multiple groups.

**Supplementary Figure 22.** (**A**) Fluorescence images and (**B**) quantitative analysis of the fluorescence intensity in the major organs and infection sites after intravenous injection of mGls@HEV-MTP for 72 h (*n =* 5). All *n* values indicated the number of independent biological replicates. All data were presented as the mean ± standard deviation. The *p*-values were determined by (**B**) one-way ANOVA with Tukey’s post hoc test for comparisons among multiple groups.

**Supplementary Figure 23.** Negative control for flow cytometry analysis of CD86^+^ CD206^-^ cells in Figure 6H.

**Supplementary Figure 24.** (**A**) Representative flow cytometry data of intracellular residual *S. aureus*, (**B**) CellROX Deep Red staining to depict intracellular ROS levels, and (**C**) MitoTracker Red CM-H2Xros staining to depict intracellular mtROS levels of infected macrophages upon different treatments at 7dpi (*n* = 5). All *n* values indicated the number of independent biological replicates. All data were presented as the mean ± standard deviation. The *p*-values were determined by (**A**-**B**) one-way ANOVA with Tukey’s post hoc test for comparisons among multiple groups.

**Supplementary Figure 25.** Representative images of major organs after indicated treatments. Scale bar, 800 μm.

**Supplementary Tables**

Supplementary Table 1. GESA analysis for RAW 264.7 infected with methicillin-resistant Staphylococcus aureus

| **Term** | **3d vs NC** | | | **2w vs NC** | | | **2w vs 3d** | | |
| --- | --- | --- | --- | --- | --- | --- | --- | --- | --- |
|  | **NES** | **P value** | **FDR** | **NES** | **P value** | **FDR** | **NES** | **P value** | **FDR** |
| Glutamine family amino acid biosynthetic process (GO:0009084) | -1.0172 | 0.4559 | 0.7655 | -1.5861 | 0.0229 | 0.0321 | -1.7283 | 0.0197 | 0.0084 |
| Glutamine family amino acid catabolic process (GO:0009065) | -0.7626 | 0.7824 | 0.7495 | -0.9886 | 0.4907 | 0.4822 | -1.2871 | 0.1581 | 0.1178 |
| Glutamine family amino acid metabolic process (GO:0009064) | 0.9879 | 0.4495 | 0.4650 | -1.4228 | 0.0419 | 0.0858 | -1.8742 | 0.0023 | 0.0143 |
| Glutamine metabolic process (GO:0006541) | -0.9500 | 0.5417 | 0.7769 | -1.3209 | 0.1211 | 0.1368 | -1.2047 | 0.2253 | 0.1571 |
| L−glutamate transmembrane transport (GO:0015813) | 0.8860 | 0.6280 | 0.4516 | -0.8202 | 0.7116 | 0.7026 | -1.1560 | 0.0147 | 0.0202 |
| Amino acid biosynthetic process (GO:0008652) | -1.0541 | 0.3722 | 0.8768 | -1.9439 | 0.0007 | 0.0024 | -2.2535 | 0.0052 | 0.0190 |
| Amino acid metabolic process (GO:0006520) | -1.1397 | 0.1739 | 0.8783 | -1.6594 | 0.0010 | 0.0197 | -1.7337 | 0.0003 | 0.0071 |
| Amino acid transmembrane transport (GO:0003333) | 0.8874 | 0.6901 | 0.6707 | -1.1067 | 0.2706 | 0.3280 | -1.4962 | 0.0165 | 0.0370 |
| Amino acid transport (GO:0006865) | -0.9317 | 0.6101 | 0.6895 | -1.2896 | 0.0647 | 0.1416 | -1.5528 | 0.0046 | 0.0253 |

Supplementary Table 2. GESA analysis in the other amino acid-related metabolic pathways for RAW 264.7 infected with methicillin-resistant Staphylococcus aureus

| **Term** | **3d vs NC** | | | **2w vs NC** | | | **2w vs 3d** | | |
| --- | --- | --- | --- | --- | --- | --- | --- | --- | --- |
|  | **NES** | **P value** | **FDR** | **NES** | **P value** | **FDR** | **NES** | **P value** | **FDR** |
| Tryptophan metabolism | 1.2523 | 0.1660 | 0.9813 | -1.0080 | 0.4385 | 0.3842 | -1.4422 | 0.0626 | 0.1860 |
| Sulfur amino acid metabolic process | 1.1112 | 0.2992 | 0.9813 | -1.4713 | 0.0340 | 0.0929 | -1.4591 | 0.0574 | 0.1860 |
| Sulfur amino acid biosynthetic process | 0.8996 | 0.6297 | 0.9813 | -1.5802 | 0.0428 | 0.0929 | -1.4203 | 0.0797 | 0.1860 |
| Aspartate family amino acid metabolic process | -0.7968 | 0.8276 | 0.9813 | -1.4041 | 0.0530 | 0.0929 | -1.1730 | 0.2336 | 0.4087 |
| Aspartate family amino acid biosynthetic process | 0.5783 | 0.9605 | 0.9813 | -0.9737 | 0.5110 | 0.3842 | -1.0586 | 0.3812 | 0.4391 |
| Branched chain amino acid metabolic process | -0.7177 | 0.8543 | 0.9813 | -1.3510 | 0.0923 | 0.0971 | -1.0136 | 0.4391 | 0.4391 |
| Methionine metabolic process | 0.4910 | 0.9813 | 0.9813 | -1.3340 | 0.0757 | 0.0971 | -1.1202 | 0.3141 | 0.4391 |

Supplementary Table 3. GESA analysis for GSE166522

| **Term** | **3d vs NC** | | | **2w vs NC** | | | **2w vs 3d** | | |
| --- | --- | --- | --- | --- | --- | --- | --- | --- | --- |
|  | **NES** | **P value** | **FDR** | **NES** | **P value** | **FDR** | **NES** | **P value** | **FDR** |
| Glutamine family amino acid biosynthetic process (GO:0009084) | 1.0754 | 0.3594 | 0.5612 | -0.7989 | 0.7145 | 0.7115 | 0.5626 | 0.9591 | 0.7408 |
| Glutamine family amino acid catabolic process (GO:0009065) | 1.2019 | 0.2318 | 0.3896 | 1.2432 | 0.1976 | 0.0917 | 0.9212 | 0.5825 | 0.9028 |
| Glutamine family amino acid metabolic process (GO:0009064) | 1.4324 | 0.0382 | 0.1454 | -0.9150 | 0.6068 | 0.6127 | -1.3524 | 0.0536 | 0.1434 |
| Glutamine metabolic process (GO:0006541) | 1.0078 | 0.4473 | 0.5057 | -1.0721 | 0.3750 | 0.3993 | -1.7192 | 0.0120 | 0.0167 |
| L−glutamate transmembrane transport (GO:0015813) | 1.0182 | 0.4275 | 0.5833 | 1.2711 | 0.1499 | 0.1189 | 0.7473 | 0.8399 | 0.8848 |
| Amino acid biosynthetic process (GO:0008652) | 0.8633 | 0.7029 | 0.6299 | -1.2307 | 0.1635 | 0.5562 | -1.2076 | 0.1540 | 0.1705 |
| Amino acid metabolic process (GO:0006520) | 0.9979 | 0.4607 | 0.4525 | -1.0807 | 0.2881 | 0.4641 | -1.0649 | 0.2764 | 0.2984 |
| Amino acid transmembrane transport (GO:0003333) | -1.1612 | 0.1843 | 0.1403 | -1.1703 | 0.1835 | 0.5059 | -1.2149 | 0.1368 | 0.1972 |
| Amino acid transport (GO:0006865) | -1.2904 | 0.0498 | 0.1039 | -1.0894 | 0.2843 | 0.5589 | -1.2367 | 0.0755 | 0.2205 |

Supplementary Table 4. Flow cytometry antibody list for this study.

| **Color** | **Antibody** | **Clone** | **Company** | **Cat.#** | **Dilution** |
| --- | --- | --- | --- | --- | --- |
| UV | Zombie | N/A | Biolegend | 423107 | 1:200 |
| APC/Cy7 | CD45 | 30-F11 | Biolegend | 103116 | 1:200 |
| BV605 | CD45 | 30-F11 | Biolegend | 103139 | 1:200 |
| BV510 | CD11b | M1/70 | Biolegend | 101245 | 1:200 |
| PE | F4/80 | BM8 | Biolegend | 123110 | 1:200 |
| FITC | CD86 | GL-1 | Biolegend | 105006 | 1:200 |
| PerCP/Cy5.5 | CD86 | GL-1 | Biolegend | 105028 | 1:200 |
| BV605 | I-A/I-E | M5/114.15.2 | Biolegend | 107639 | 1:200 |
| APC | CD206 | C068C2 | Biolegend | 141708 | 1:200 |
| PE | CD206 | C068C2 | Biolegend | 141706 | 1:200 |
| PerCP/Cy5.5 | PD-L1 | 10F.9G2 | Biolegend | 124334 | 1:200 |
| BV421 | CD3 | 17A2 | Biolegend | 100228 | 1:200 |
| FITC | CD4 | GK1.5 | Biolegend | 100405 | 1:200 |
| PE/dazzle 594 | CD11c | N418 | Biolegend | 117348 | 1:200 |
| PE | CD8a | 53-6.7 | Biolegend | 100707 | 1:200 |
| BV785 | Ly6G | 1A8 | Biolegend | 127645 | 1:200 |
| PE/Cy7 | Ly6C | HK1.4 | Biolegend | 128018 | 1:200 |
| PerCP/Cy5.5 | CD63 | NVG-2 | Biolegend | 143911 | 1:200 |
| PE-Cy7 | iNOS | CXNFT | Invitrogen | 25-5920-80 | 1:200 |
| N/A | CD16/32 | S17011E | Biolegend | 156604 | 1:100 |

N/A, Not Applicable.

Supplementary Table 5. Primer sequence for RT-PCR analysis.

| **Gene ID** | **Protein product** | **Primer** | **Sequences** |
| --- | --- | --- | --- |
| Gls | Glutaminase (GLS) | Forward Primer | GCACTACACTTTGGACACCA |
|  |  | Reverse Primer | TAGCAACCCGTCGAGATT |
| Ccr7 | C-C chemokine receptor 7 (CCR7) | Forward Primer | CAGGTGTGCTTCTGCCAAGAT |
|  |  | Reverse Primer | GGTAGGTATCCGTCATGGTCT |
| Tnfa | Tumor necrosis factor α (TNF-α) | Forward Primer | CAGGCGGTGCCTATGTCTC |
|  |  | Reverse Primer | CGATCACCCCGAAGTTCAGTAG |
| Il1b | Interleukin 1β (IL-1β) | Forward Primer | GAAATGCCACCTTTTGACAGTG |
|  |  | Reverse Primer | TGGATGCTCTCATCAGGACAG |
| Il6 | Interleukin 6 (IL-6) | Forward Primer | CTGCAAGAGACTTCCATCCAG |
|  |  | Reverse Primer | AGTGGTATAGACAGGTCTGTTGG |
| Il10 | Interleukin 10 (IL-10) | Forward Primer | CTTACTGACTGGCATGAGGATCA |
|  |  | Reverse Primer | GCAGCTCTAGGAGCATGTGG |
| Ccl2 | Chemokine C-C motif ligand 2 (CCL2) | Forward Primer | TAAAAACCTGGATCGGAACCAAA |
|  |  | Reverse Primer | GCATTAGCTTCAGATTTACGGGT |
| Cxcl1 | Chemokine (C-X-C motif) ligand 1 (CXCL1) | Forward Primer | CCGAAGTCATAGCCACACTCAA |
|  |  | Reverse Primer | GCAGTCTGTCTTCTTTCTCCGTTA |
| Cxcl2 | Chemokine (C-X-C motif) ligand 2 (CXCL2) | Forward Primer | GAAGTCATAGCCACTCTCAAGG |
|  |  | Reverse Primer | CCTCCTTTCCAGGTCAGTTAGC |
| actb | actin | Forward Primer | TCCATCATGAAGTGTGACGT |
|  |  | Reverse Primer | TACTCCTGCTTGCTGATCCAC |

N/A, Not Applicable.

Supplementary Table 6. Small molecules used in the study.

| **Compound** | **Concentration used (tested)** | **Toxic effects observed** | **Company (Cat.#)** |
| --- | --- | --- | --- |
| BPTES | 5 (2-5) μM | No toxic effects observed for the concentrations tested over 7 days | MCE (HY-12683) |
| Dimethyl-α-ketoglutarate (DM-αKG) | 1 (0.5-2) mM | No toxic effects observed for the concentrations tested over 7 days | Sigma-Aldrich (349631) |
| GSK2795039 | 25 (5-30) μM | No toxic effects observed for the concentrations tested over 7 days | MCE (HY-18950) |
| MitoTEMPO^19-21^ | 100 (100-300) μM | No toxic effects observed for the concentrations tested over 7 days | Sigma-Aldrich (SML0737) |

Supplementary Table 7. West blot antibodies for this study.

|  | **Antibody** | **Cat.** | **Company, Country** |
| --- | --- | --- | --- |
| Primary antibodies | GLS1 | 12855-1-AP | Proteintech, China |
|  | β-actin | 66009-1-Ig | Proteintech, China |
| Second antibodies | HRP Conjugated AffiniPure Goat Anti-rabbit IgG (H+L) | BA1055 | Boster, China |
|  | HRP Conjugated AffiniPure Goat Anti-mouse IgG (H+L) | BA1051 | Boster, China |

Supplementary Table 8. Gross bone pathological score for infected bone tissue and osteomyelitis^22^.

| Macroscopic feature | Score |
| --- | --- |
| No abscess, sequestrum, active bone formation and erythema | 0 |
| Minimal erythema without abscess or evidence of new bone formation | 1 |
| Erythema with widening of the head and shaft of the bone with new bone formation | 2 |
| Abscess with new bone formation, sinus tract drainage, or grossly purulent exudate | 3 |
| Severe bone resorption, abscess and diaphyseal or total tibial involvement | 4 |

Supplementary Table 9. Smeltzer's scoring system for infected bone tissue and osteomyelitis^23^.

| Histological parameter | Score |
| --- | --- |
| I. Intraosseous acute inflammation |  |
| Not present | 0 |
| Minimal to mild inflammation with no intramedullary abscess | 1 |
| Moderate to severe inflammation with no intramedullary abscess | 2 |
| Minimal to mild inflammation with intramedullary abscess | 3 |
| Moderate to severe inflammation with intramedullary abscess | 4 |
| II. Intraosseous chronic inflammation |  |
| Not present | 0 |
| Minimal to mild chronic inflammation with no significant intramedullary fibrosis | 1 |
| Moderate to severe inflammation with no significant intramedullary fibrosis | 2 |
| Minimal to mild inflammation with significant intramedullary fibrosis | 3 |
| Moderate to severe inflammation with significant intramedullary fibrosis | 4 |
| III. Periosteal inflammation |  |
| Not present | 0 |
| Minimal to mild inflammation with no subperiosteal abscess formation | 1 |
| Moderate to severe inflammation with no subperiosteal abscess formation | 2 |
| Minimal to mild inflammation with subperiosteal abscess formation | 3 |
| Moderate to severe inflammation with subperiosteal abscess formation | 4 |
| IV. Bone necrosis |  |
| No evidence of necrosis | 0 |
| Single focus of necrosis without sequestrum formation | 1 |
| multiple foci of necrosis without sequestrum formation | 2 |
| Single focus of sequestrum | 3 |
| multiple foci sequestra | 4 |
| Total scores |  |
| Significant infection | ≥10 |

**References**

1.	Zhang X, Cao D, Xu L, Xu Y, Gao Z, Pan Y, et al. Harnessing matrix stiffness to engineer a bone marrow niche for hematopoietic stem cell rejuvenation. Cell Stem Cell **30**, 378-395 (2023).

2.	Qiao Y, Liu X, Li B, Han Y, Zheng Y, Yeung KWK, et al. Treatment of MRSA-infected osteomyelitis using bacterial capturing, magnetically targeted composites with microwave-assisted bacterial killing. Nat. Commu. **11**, 4446 (2020).

3.	Chen L, Shao Z, Zhang Z, Teng W, Mou H, Jin X, et al. An On-Demand Collaborative Innate-Adaptive Immune Response to Infection Treatment. Adv. Mater. **36**, e2304774 (2024).

4.	Lin Y, Su J, Wang Y, Xu D, Zhang X, Yu B. mRNA Transcriptome Analysis of Bone in a Mouse Model of Implant-Associated Staphylococcus aureus Osteomyelitis. Infect. Immun. **89**, e00814-00820 (2021).

5.	Gu Z, Eils R, Schlesner M. Complex heatmaps reveal patterns and correlations in multidimensional genomic data. Bioinformatics **32**, 2847-2849 (2016).

6.	Wu T, Hu E, Xu S, Chen M, Guo P, Dai Z, et al. clusterProfiler 4.0: A universal enrichment tool for interpreting omics data. Innovation **2**, 100141 (2021).

7.	Hänzelmann S, Castelo R, Guinney J. GSVA: gene set variation analysis for microarray and RNA-seq data. BMC Bioinformatics **14**, 7 (2013).

8.	Horn CM, Arumugam P, Van Roy Z, Heim CE, Fallet RW, Bertrand BP, et al. Granulocytic myeloid-derived suppressor cell activity during biofilm infection is regulated by a glycolysis/HIF1a axis. J. Clin. Invest. **134**, e174051 (2024).

9.	Hao Y, Hao S, Andersen-Nissen E, Mauck WM, Zheng S, Butler A, et al. Integrated analysis of multimodal single-cell data. Cell **184**, 3573-3587 (2021).

10.	Korsunsky I, Millard N, Fan J, Slowikowski K, Zhang F, Wei K, et al. Fast, sensitive and accurate integration of single-cell data with Harmony. Nat. Methods **16**, 1289-1296 (2019).

11.	Aibar S, González-Blas CB, Moerman T, Huynh-Thu VA, Imrichova H, Hulselmans G, et al. SCENIC: single-cell regulatory network inference and clustering. Nat. Methods **14**, 1083-1086 (2017).

12.	Caire R, Audoux E, Thomas M, Dalix E, Peyron A, Rodriguez K, et al. YAP promotes cell-autonomous immune responses to tackle intracellular Staphylococcus aureus in vitro. Nat. Commu. **13**, 6995 (2022).

13.	Rodrigues Lopes I, Alcantara LM, Silva RJ, Josse J, Vega EP, Cabrerizo AM, et al. Microscopy-based phenotypic profiling of infection by Staphylococcus aureus clinical isolates reveals intracellular lifestyle as a prevalent feature. Nat. Commu. **13**, 7174 (2022).

14.	Peyrusson F, Varet H, Nguyen TK, Legendre R, Sismeiro O, Coppée J-Y, et al. Intracellular Staphylococcus aureus persisters upon antibiotic exposure. Nat. Commu. **11**, 2200 (2020).

15.	Verbeke R, Hogan MJ, Loré K, Pardi N. Innate immune mechanisms of mRNA vaccines. Immunity **55**, 1993-2005 (2022).

16.	Brook B, Duval V, Barman S, Speciner L, Sweitzer C, Khanmohammed A, et al. Adjuvantation of a SARS-CoV-2 mRNA vaccine with controlled tissue-specific expression of an mRNA encoding IL-12p70. Sci. Transl. Med. **16**, eadm8451 (2024).

17.	Xia Y, Sokhi UK, Bell RD, Pannellini T, Turajane K, Niu Y, et al. Immune and repair responses in joint tissues and lymph nodes after knee arthroplasty surgery in mice. Journal of Bone and Mineral Research : the Official Journal of the American Society For Bone and Mineral Research **36**, 1765-1780 (2021).

18.	Xuan Q, Li H, Gao Y, Qiao X, Feng Y, Yu X, et al. Photo-reversible amyloid nanoNETs for regenerative antimicrobial therapies. Nat. Commu. **16**, 11025 (2025).

19.	Mills EL, Kelly B, Logan A, Costa ASH, Varma M, Bryant CE, et al. Succinate Dehydrogenase Supports Metabolic Repurposing of Mitochondria to Drive Inflammatory Macrophages. Cell **167**, (2016).

20.	Guan X, Yang B, Bai B, Cui N, Wang M, Su J, et al. Manganese enhances macrophage bactericidal activity in mice with Staphylococcus aureus osteomyelitis via Sirt3-mitophagy axis. Nat. Commu., (2026).

21.	Zotta A, Toller-Kawahisa J, Palsson-McDermott EM, O'Carroll SM, Henry ÓC, Day EA, et al. Mitochondrial respiratory complex III sustains IL-10 production in activated macrophages and promotes tumor-mediated immune evasion. Sci. Adv. **11**, eadq7307 (2025).

22.	Rissing JP, Buxton TB, Weinstein RS, Shockley RK. Model of experimental chronic osteomyelitis in rats. Infect. Immun. **47**, 581-586 (1985).

23.	Smeltzer MS, Thomas JR, Hickmon SG, Skinner RA, Nelson CL, Griffith D, et al. Characterization of a rabbit model of staphylococcal osteomyelitis. J. Orthop. Res. **15**, 414-421 (1997).
